# FDA-Approved Fluorinated Heterocyclic Drugs from 2016 to 2022

**DOI:** 10.3390/ijms24097728

**Published:** 2023-04-23

**Authors:** Carla Rizzo, Sara Amata, Ivana Pibiri, Andrea Pace, Silvestre Buscemi, Antonio Palumbo Piccionello

**Affiliations:** Department of Biological, Chemical and Pharmaceutical Sciences and Technologies (STEBICEF), University of Palermo, Viale delle Scienze, Ed. 17, 90128 Palermo, Italy; carla.rizzo03@unipa.it (C.R.); sara.amata01@unipa.it (S.A.); ivana.pibiri@unipa.it (I.P.); andrea.pace@unipa.it (A.P.); silvestre.buscemi@unipa.it (S.B.)

**Keywords:** FDA-approved, fluorine, heterocycles

## Abstract

The inclusion of fluorine atoms or heterocyclic moiety into drug structures represents a recurrent motif in medicinal chemistry. The combination of these two features is constantly appearing in new molecular entities with various biological activities. This is demonstrated by the increasing number of newly synthesized fluorinated heterocyclic compounds among the Food and Drug Administration FDA-approved drugs. In this review, the biological activity, as well as the synthetic aspects, of 33 recently FDA-approved fluorinated heterocyclic drugs from 2016 to 2022 are highlighted.

## 1. Introduction

The presence of many different heterocyclic rings in natural products, such as alkaloids, vitamins, antibiotics, peptides, etc., prompted the introduction of structural motifs into synthetic drugs [1,2].

Therefore, heterocycles are considered to be prominent scaffolds for the synthesis of biologically active compounds and prospective drugs [3,4]. In fact, it is estimated that heterocyclic moieties are present in around 85% of bioactive compounds [5]. On the other hand, in the second half of 20th century, another fundamental tool for drug design was introduced with the incorporation of fluorine atoms into drugs [6,7,8]. Since the introduction of the first fluorocorticosteroid, fludrocortisone, in 1954 [9], the fluorinated drugs market has exponentially evolved, with 20% of those on the market being fluorinated drugs and around 30% of fluorinated drugs being blockbuster pharmaceuticals, such as Lipitor, Fluoxetine, Linezolid or Fluticasone [8]. To date, more than 300 fluorinated pharmaceuticals have been approved for use as drugs [6]. The success of the introduction of fluorine atoms is linked to the peculiar physicochemical properties of the C-F bond [10], which are the high bond strength, polarity and minimal steric hindrance of fluorine [11], combined with a general metabolic stability that, nevertheless, is an issue that is currently under exploration [12].

The introduction of fluorine, for example, allows researchers to easily modulate the p*K*_a_ of neighboring functionalities, improving the bioavailability and affinity to specific receptors [13,14].

In addition, the monofluorination or trifluoromethylation of alkyl groups decreases the drug lipophilicity due to the strong electron-withdrawing capabilities of fluorine. On the other hand, fluoro-arenes are more lipophilic due to the low polarizability of the C-F bond [15]. In addition, the presence of a fluorine atom can also enhance the membrane’s permeability [16].

The importance of fluorinated compounds is also linked to their use as diagnostic tools within imaging techniques such as ^19^F-MRI and ^18^F-PET [17,18]. 

**Figure 1:**
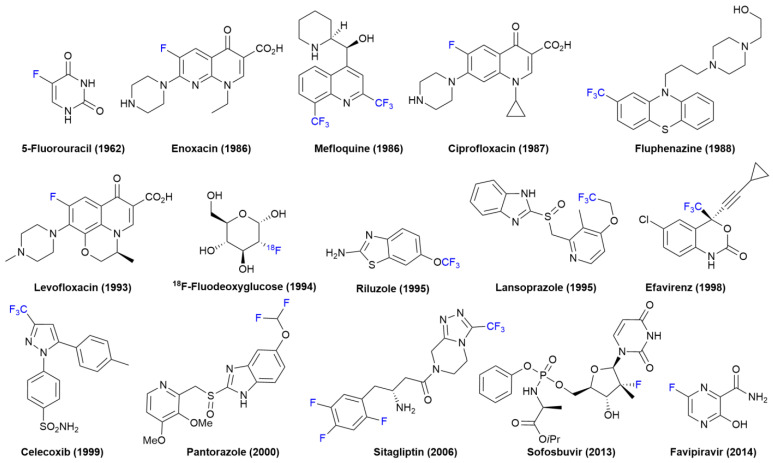
Structures of representative fluorinated heterocyclic drugs approved by FDA, with the year of approval in brackets.

The direct link between fluorinated moieties and heterocycles led to the formation of the sub-class of fluorinated heterocycles, which combine the strength of these two fundamental scaffolds in modern medicinal chemistry. This important class of fluorinated pharmaceuticals includes some of the selected examples of FDA-approved drugs reported in Figure 1. Among these compounds, there have been several game changers over the last decades, such as fluorouracil, the class of fluoroquinolones, sitagliptin and fluorodeoxyglucose, just to mention a few [19].

In this review, recent advances in the field of fluorinated heterocyclic drugs are presented, discussing FDA-approved molecules from 2016 to 2022. The molecules considered in this article are limited to those with a fluorinated group directly linked to the heterocyclic ring. The biological targets and the therapeutic indications are presented together with synthetic details. The fluorination strategy and influence of the fluorinated moiety on bioactivity are also discussed. The sections are reported in chronological order, starting with the most recently approved drugs; for each section, the compounds are presented in alphabetical order.

### Introduction of Fluorine Atoms in Organic Molecules

Fluorinated starting materials used as precursors to obtain fluorinated, approved drugs can present mono-, di- and trifluoro alkyl groups; the last ones can be generally introduced via building blocks such as trifluoroacetate, trifluoroethylamine, trifluoro and ethyl triflate. Furthermore, several starting materials used for this purpose are formed by aromatic or heterocyclic rings bearing F atoms, such as (poly)fluorobenzoic acid, fluoro- or trifluoromethylpyridines, just to cite a few of them (see below). 

**Figure 2:**
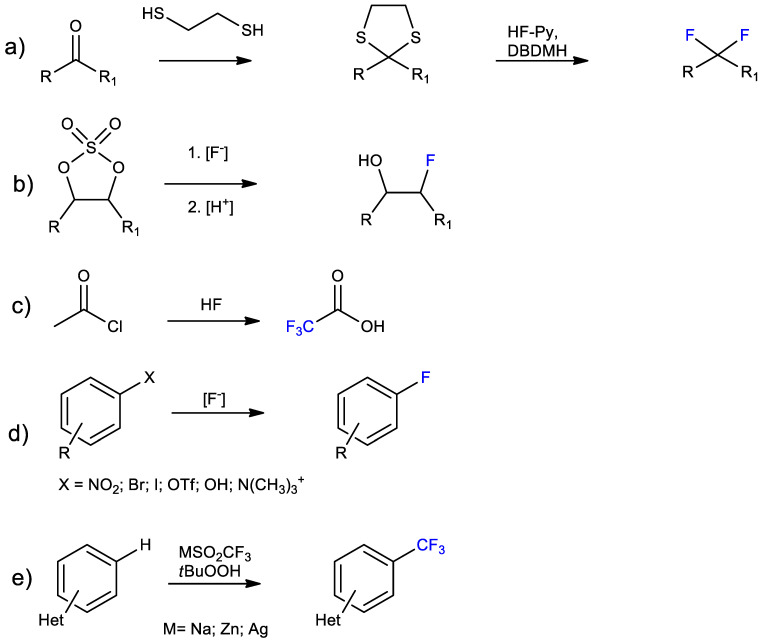
Representative reaction schemes for introduction of F atoms into organic molecules. Introduction of (**a**) two, (**b**) one or (**c**) three fluorine atoms into alkyl chains. Introduction of (**d**) one fluorine or (**e**) trifluoromethyl into aromatics.

Some of the main processes used for the introduction of F atoms are summarized in Figure 2. The introduction of C-F or CF_2_ groups is achieved through the nucleophilic fluorination of electrophiles; some examples involving dithiane or sulphonate formation are reported in Figure 2a,b [20,21]. 

Trifluoromethylated starting materials such as trifluoroacetic acid are industrially prepared in excellent yields by the electrochemical fluorination of acetyl chloride or acetic anhydride in anhydrous hydrogen fluoride, followed by the hydrolysis of the resulting trifluoroacetyl fluoride (Figure 2c) [22]. Fluorination methods of arenes include traditional nucleophilic substitution (Figure 2d) and transition-metal-catalyzed nucleophilic fluorination or deoxofluorination [21]. For the introduction of trifluoromethyl groups, substrates can be trifluoromethylated by employing electrophilic trifluoromethylating reagents [23], such as Togni reagents [24], and S-(trifluoromethyl)dibenzothiophenium salts [25], or lower cost reagents such as CF_3_I or CF_3_H, which are favored for industrial processes, while the use of the solid and bench-top-stable reagents such as NaSO_2_CF_3_ in radical trifluoromethylations for the trifluoromethylation of electron-rich arenes avoids perfluoroalkylations (Figure 2e) [21].

## 2. FDA-Approved Drugs in 2022

**Scheme 1:**
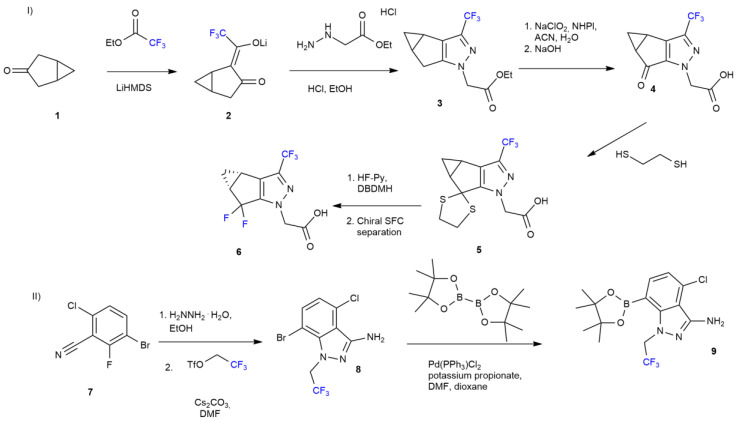
Synthesis of Lenacapavir, part (**I–II**). (**I**) Synthesis of intermediate **6**; (**II**) Synthesis of intermediate **9**.

**Scheme 2:**
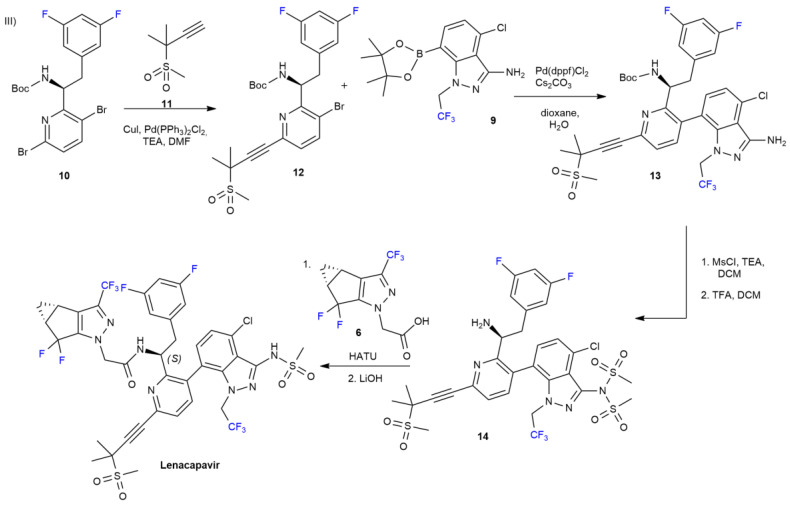
Synthesis of Lenacapavir, part (**III**).

**Scheme 3:**
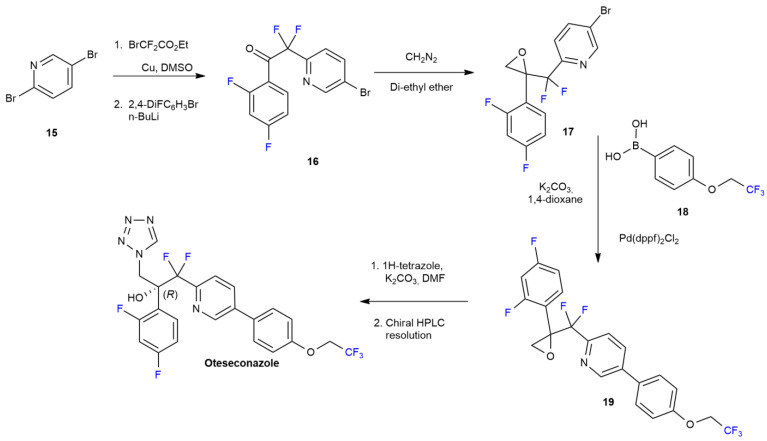
Synthesis of Oteseconazole.

In 2022, the FDA approved 37 new therapeutic and diagnostic products [26]. Monoclonal antibodies (mAbs) continue to be one of the most widely licensed groups of biological therapies. Nevertheless, 22 of them are novel chemical entities (NCEs), 14 of which contain fluorine atoms and nitrogen heterocycles [27]. ***Lenacapavir*** (Scheme 1 and Scheme 2) and ***Oteseconazole*** (Scheme 3), two new approved drugs released in the past year, combine these two key characteristics.

### 2.1. Lenacapavir

***Lenacapavir*** (SUNLENCA^®^) is a human immunodeficiency virus type 1 (HIV-1) capsid inhibitor developed by Gilead Science Inc., and it is administered in cases when conventional antiretroviral therapies are ineffective. The mechanism of action is totally different from that of other antivirals used for the treatment of HIV-1. Indeed, ***Lenacapavir*** establishes several hydrophobic and electrostatic interactions with capsid subunits (CA1 and CA2). For example, the difluorobenzyl group can be stabilized inside a hydrophobic pocket of the CA1 *N*-terminal domain. This entails interference with virus life cycle processes where CA is involved, such as reverse transcription, nuclear import and integration [28,29]. 

The synthetic method to obtain ***Lenacapavir*** is divided into three steps and is reported in Scheme 1 and Scheme 2 [30].

The Claisen condensation of **1** using a strong base, such as lithium bis(trimethylsilyl)amide and ethyl 2,2,2-trifluoroacetate, leads to enolate **2**, presenting a CF_3_ group. At this point, pyrazole ring **3** formation occurs due to the addition of an ethyl hydrazinoacetate salt. 

Intermediate **4** is obtained through *N*-hydroxyphtalimide-catalyzed selective oxidation, followed by saponification with NaOH. To obtain building block **6**, the desulfurative fluorination of dithiolane **5** takes place, followed by supercritical fluid chromatography (SFC).

The synthesis proceeds with the construction of building block **9**. To acquire substituted indazole core **8**, hydrazine is combined with fluorobenzonitrile **7**, and then a trifluoroethyl group is introduced via a substitution at position one.

A cross-coupling reaction between bis(pinacolato)diboron and **8** in the presence of a palladium/triphenylphosphine catalyst and potassium propionate creates **9**. In order to produce ***Lenacapavir***, deprotection preceded by the formation of an amide bond between carboxylic acid **6** and ammine **14** must occur. 

Intermediate **14** is obtained via two palladium-catalyzed coupling reactions between fluorinated compound **10** and amine **13**, followed by a protection with methanesulfonyl chloride on the amino group linked to the indazole ring.

### 2.2. Oteseconazole 

***Oteseconazole*** (VIVJOA™) is an antifungal agent that was released by Mycovia Pharmaceuticals and is administered to reduce the incidence of recurrent vulvovaginal candidiasis (RVVC). It affects the integrity of the cell membrane of pathogenic strains of candida by interacting with cytochrome P450 (CYP51) [31,32].

***Oteseconazole***’s selectivity for fungal metalloenzyme CYP51 is provided by the tetrazole moiety. In turn, the heterocyclic residue is connected through a metabolically resistant difluoro methyl linker with a substituted phenyl trifluoroethyl ether. The synthesis is presented in Scheme 3 [33].

Starting with pyridine **15**, ethyl bromodifluoroacetate and 2,4-difluorobromobenzene are used to introduce the CF_2_ linker and 2,4-difluorobenzene group, respectively, producing **16**. Through a diazomethane-mediated epoxidation reaction, intermediate **17** is obtained and reacts with 4-(trifluoromethoxy)phenylboronic acid **18** via a Pd catalyzed Suzuky–Miyaura coupling reaction, producing **19**. In the next two steps, the introduction of the triazole ring occurs via nucleophilic attack, leading to the opening of the epoxy ring. ***Oteseconazole*** is finally achieved as a single enantiomer by chiral preparative HPLC.

## 3. FDA-Approved Drugs in 2021

**Scheme 4:**
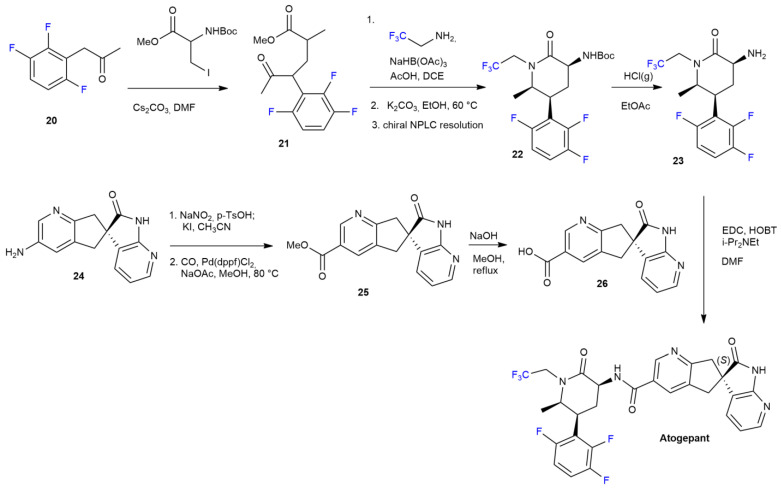
Synthesis of Apogent.

**Scheme 5:**
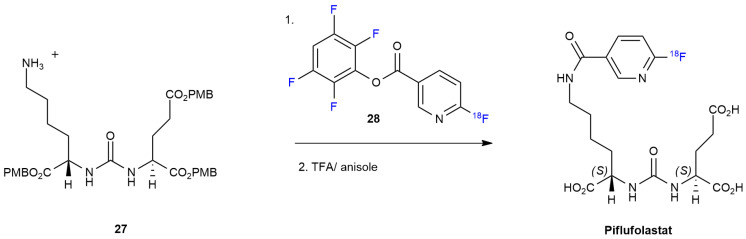
Synthesis of Piflufolast ^18^F.

**Scheme 6:**
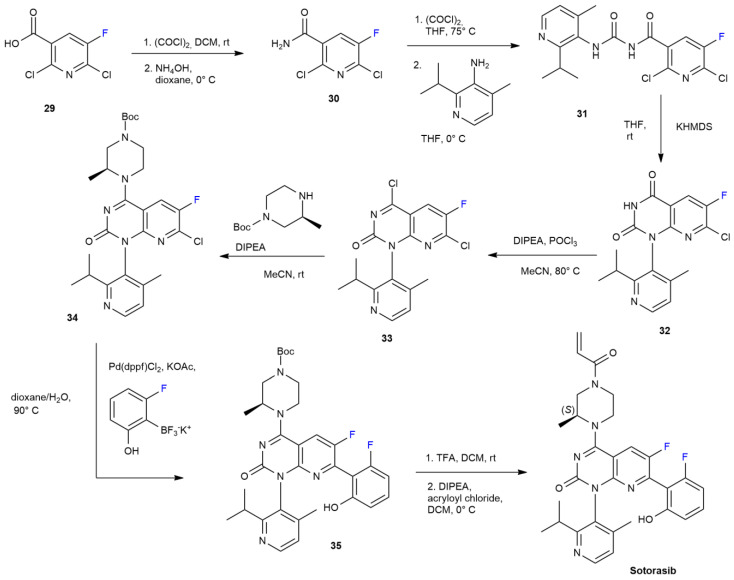
Synthesis of Sotorasib.

**Scheme 7:**
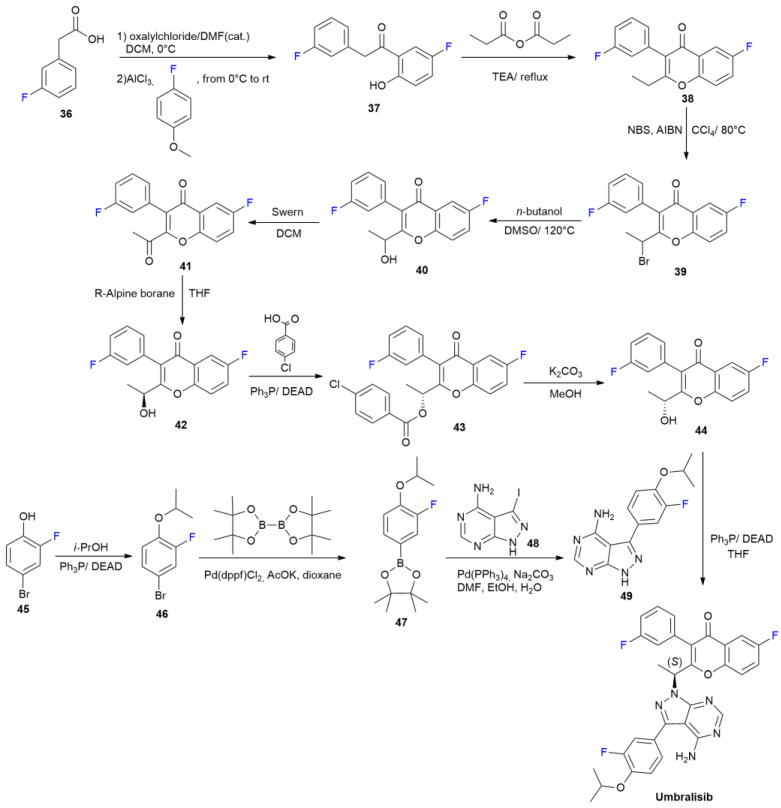
Synthesis of Umbralisib.

**Scheme 8:**
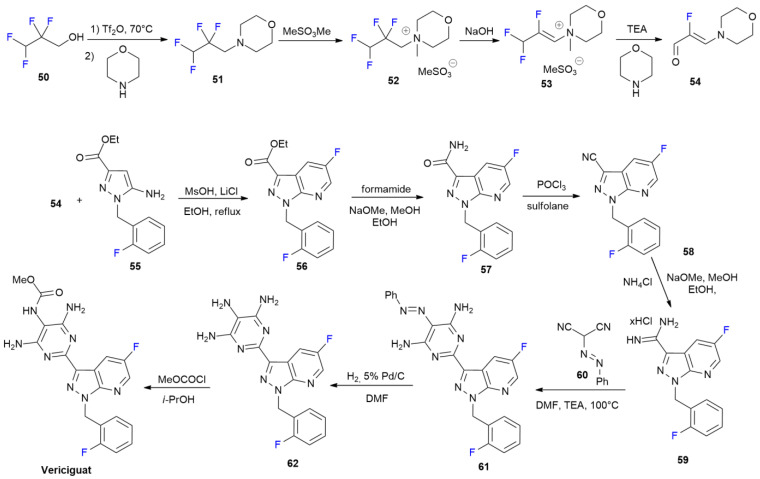
Synthesis of Vericiguat.

In 2021, 50 new drugs were approved. Thirty-three small molecules with 10 fluorinated compounds and 28 heterocyclic compounds are included in this list, together with fluorinated heterocycles ***Atogepant***, ***Piflufolastat***, ***Sotorasib***, ***Umbralisib***, and ***Vericiguat*** (Scheme 4, Scheme 5, Scheme 6, Scheme 7 and Scheme 8), as discussed below [34].

### 3.1. Atogepant

***Atogepant*** (Qulipta™) is a novel drug designed by Abb Vie for the preventive treatment of migraines in adults. Developed as a calcitonin gene-related peptide (CGRP) receptor antagonist, this neuropeptide and its receptors are located in the trigeminal nerves involved in pain sensations [35]. The antagonist’s high affinity to the receptor is increased due to a 2,3,6 fluoro substitution on the phenyl moiety. The 2,2,2-trifluoethyl group masking the piperidinone ring improved the pharmacokinetic and pharmacodynamic characteristics more than other gepant drugs do. In addition, fluorine atoms could also be associated with a lower hepatotoxicity [36]. The method for ***Atogepant*** synthesis is reported in Scheme 4 [37]. 1-(2,3,6-trifluorophenyl)propan-2-one **20** is alkylated to **21** via N-Boc-iodoserine-OMe. Piperidine intermediate **22** is a result of a reductive amination with 2,2,2-trifluoroethanamine and sodium triacetoxyborohydride as a reducing agent, followed by cyclization and optical resolution using normal-phase liquid chromatography (NPLC). In order to generate the first building block, **23**, deprotection of the amine group via hydrochloric acid takes place. The second intermediate, **26**, is achieved by performing a previously patented procedure on **24** [38]. Azospiro bispyridine **24** undergoes diazotation–iodination process via sodium nitrite in the presence of *p*-toluensulfonic acid and potassium iodide. Ester **25**, obtained via palladium-catalyzed carbonylation, is subsequently saponified to obtain **26**. Finally, a coupling reaction with aminopiperidinone **23** and carboxylic acid **26** is carried out to allow the formation of an amide bond leading to the final product, ***Atogepant.***

### 3.2. Piflufolastat F 18

***Piflufolastat*** ***F 18***, commercially known as PYLARIFY by Progenics Pharmaceuticals Inc., is a diagnostic imaging agent radiolabeled with ^18^F isotope, which detects prostate-specific membrane antigen (PSMA) via positron emission tomography (PET). It was approved on May 2021 by the FDA as a radioactive diagnostic tool, thanks to which it is possible to obtain accurate and early information on prostate cancer metastases, even in those patients with low prostate-specific antigen (PSA) levels [39,40]. 

***Piflufolastat*** synthesis is described in Scheme 5 [40].

***Piflufolastat*** is the result of the deprotection of the carboxyl group with TFA, preceded by a nucleophilic substitution with urea derivate **27** on 6-[^18^F]Fluoro-nicotinic acid-2,3,5,6-tetrafluoro-phenyl ester **28**, synthetized according to the previously reported procedure [41]. 

### 3.3. Sotorasib

The brand name for the new drug marketed by Amgen is LUMAKRASTM^TM^. The pharmacologically active agent, ***Sotorasib***, it is an RAS small GTPase inhibitor used to treat colorectal cancer and non-small-cell lung cancer brought on by the KRAS^G12C^ oncogene [42]. To improve the pharmacokinetic properties such as oral bioavailability, azaquinazolinone is designed with a fluorine atom on C6 carbon, a fluorophenol residue on C7 and a nitrogen atom instead of C8 carbon [43]. The ***Sotorasib*** synthetic process is described in Scheme 6 [44]. 

Starting with 2,6-dichloro-5-fluoronicotinic acid **29**, acyl chloride is obtained through the use of oxalyl chloride; this is then converted to the corresponding amide, **30**. The formation of compound **31** is carried out through the reaction between nicotinamide **30** and 2-isopropyl-4-methylpyridin-3-amine. Compound **31** is then treated with potassium hexamethyldisilazane to drive cyclization and produce the duly substituted 2,4-dihydroxypyrido [2,3-d]pyrimidine ring **32.** At this point, the chlorination reaction produces intermediate **33**, which, in turn, can be combined with a Boc-protected methylpiperazine to produce a selective amination. The resulting compound **34** is reacted with organotrifluoroborate salt via a Suzuki−Miyaura cross-coupling reaction to attach fluorophenol moiety **35**. Deprotection and amidation of nitrogen of the piperazine ring eventually produces ***Sotorasib***. In 2022, the same Amgen group developed a commercial manufacturing process, in which they improved several synthetical steps, starting with compound **32** [45].

### 3.4. Umbralisib

***Umbralisib*** is sold under the brand name Ukuoniq and was developed by TG Therapeutics. It was approved in February 2021 for the treatment of marginal zone lymphoma (MZL) and follicular lymphoma (FL) [46]. The mode of action of ***Umbralisib*** is related to the inhibition of kinase PI3K-delta and casein kinase CK1-epsilon. ***Umbralisib*** contains a 6-fluoro-chromen-4-one central heterocyclic core and two other fluorophenyl groups. The synthesis was disclosed in a patent in 2014 and is presented in Scheme 7 [47]. Fluorinated chromen-4-one ring **38** is constructed, starting with 3-fluorophenylacetic acid **36**, which, after conversion into chloride and subsequent acylation of 4-fluoroanisole, yields compound **37**. The treatment of phenol **37** with propionic anhydride produces **38** via acylation and subsequent cyclocondensation. The radical bromination of the methylene group with NBS yields **39**. The following steps result in the obtainment of racemic alcohol **40** after the hydrolysis of **39**, as well as the subsequent formation of two enantiomers, **42** and **44**. *S* enantiomer **42** could be selectively obtained via the stereoselective reduction of ketone **41** with *R* Alpine borane, which is obtained by means of the Swern oxidation of racemic **40**. *R* enantiomer **44** was obtained via a Mitsunobu reaction with 4-chlorobenzoic acid and DEAD, followed by the hydrolysis of ester **43**. Alcohol **44** is coupled with pyrazolopyrimidine **49**, again under Mitsunobu conditions, to acquire ***Umbralisib*** as a single enantiomer. Compound **49** is obtained via the Suzuki coupling of iodopyrazolopyrimidine **48** with aryl pinacolborane **47**. Compound **47** is obtained in two steps using 4-bromo-2-fluoro-phenol **45**.

### 3.5. Vericiguat

***Vericiguat*** is sold under the brand name Verquvo and was developed by Bayer AG and Merck & Co. It was approved in January 2021 to reduce the risks of cardiovascular death and heart failure [48].

***Vericiguat*** is a guanylate cyclase (sGC) stimulator with a *1H*-pyrazolo [3,4-b]pyridine core bearing a fluorine atom at C-5. The presence of the fluorine atom increases the metabolic stability and induces lower clearance. The method for the synthesis of ***Vericiguat*** is reported in Scheme 8 [48].

Tetrafluoropropanol **50**, the starting fluorinated building block, is converted in two steps into morpholino derivative **51**, and then into morpholinium cation **52** after methylation with methyl methanesulfonate. Compound **53** is obtained after a treatment with NaOH, which induces the elimination of the first fluorine atom as HF.

Other two fluorine atoms from the difluoromethyl group are lost during hydrolysis into 2-fluoroacrolein derivative **54**. α,β-unsaturated aldehyde **54** reacts with aminopyrazole **55** under acidic cyclization conditions, allowing the introduction of the 5-fluorine atom into the *1H*-pyrazolo [3,4-b]pyridine core of derivative **56**. Ester **56** is then converted in three steps into amidine **59**, via amide **57** and nitrile **58**. The C-3 pyrimidine ring is then constructed with a condensation between **59** and hydrazonomalonitrile **60**. Using compound **61**, the synthesis of ***Vericiguat*** is completed in two steps via the catalytic hydrogenation of the diazo moiety of **61** to triaminopyrimidine **62**, and finally, via the formation of the carbamate group of the target compound after a treatment with methyl chloroformate.

## 4. FDA-Approved Drugs in 2020

**Scheme 9:**
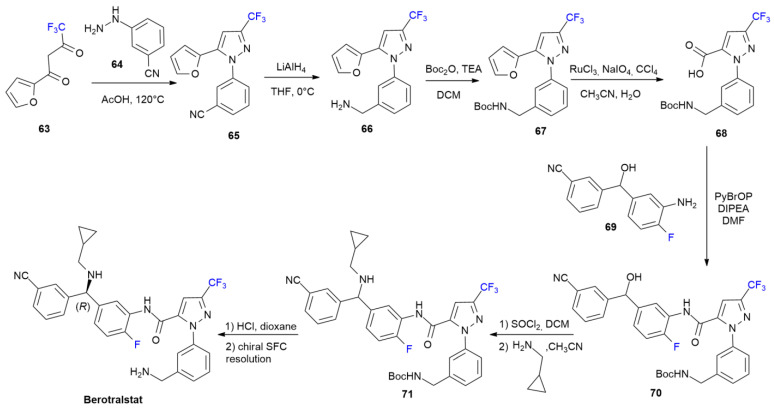
Synthesis of Berotralstat.

**Scheme 10:**
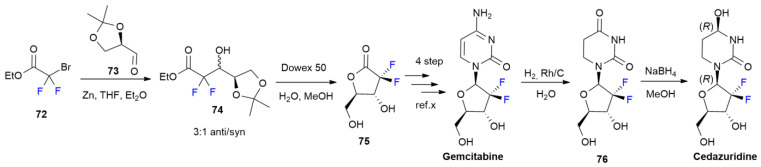
Synthesis of Cedazuridine.

**Scheme 11:**
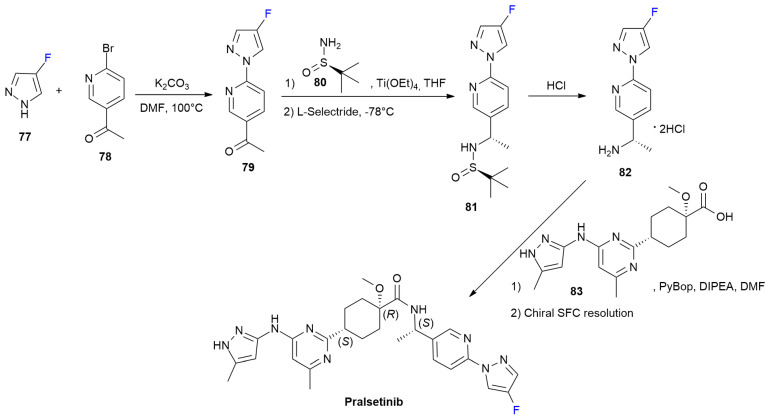
Synthesis of Pralsetinib.

**Scheme 12:**
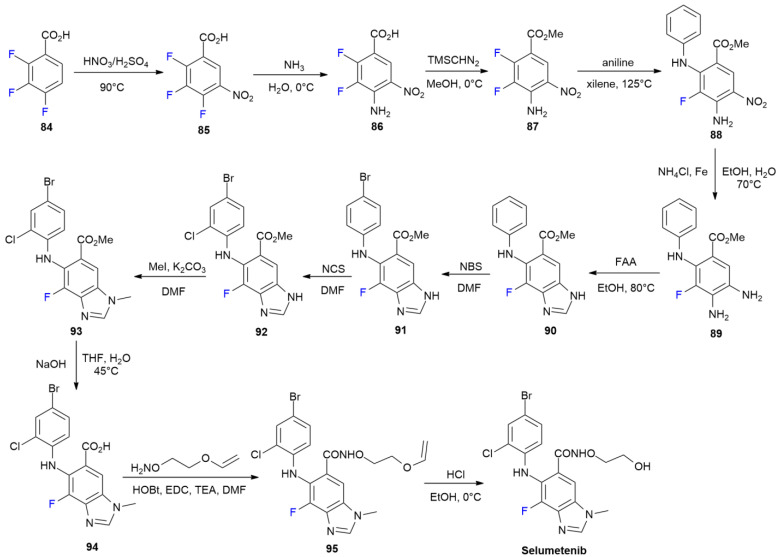
Synthesis of Selumetinib.

**Scheme 13:**
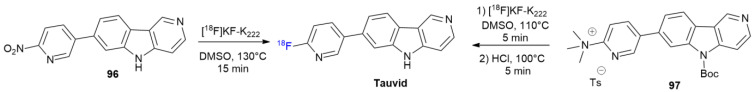
Synthesis of Tauvid.

In 2020, the FDA approved 53 new molecular entities, including 34 small molecules and 4 diagnostic agents [49]. Thirty-one out of thirty-four molecules contain at least one heterocyclic ring, and eleven out of thirty-four molecules contain at least one fluorine atom. In the following paragraph, four heterocyclic compounds bearing a fluorinated moiety directly linked to the ring are reported (Scheme 9, Scheme 10, Scheme 11 and Scheme 12). Additionally, the approved ^18^F-containing diagnostic agent, ***Tauvid***, is presented (Scheme 13).

### 4.1. Berotralstat

***Berotralstat*** is sold under the brand name Orladeyo and was developed by BioCryst Pharmaceuticals. It was approved in December 2020 to treat Hereditary Angioedema (HAE) attacks [50]. ***Berotralstat*** is a selective inhibitor of plasma kallikrein, bearing a trifluoromethylpyrazole moiety. Another fluorine is present on the central phenyl ring. The patented synthetic approach to acquiring this drug is reported below (Scheme 9) [51].

The trifluoromethylpyrazole portion of compound **65** is constructed through the condensation of trifluoro β-diketone **63** and arylhydrazine **64** in acetic acid. Cyanopyrazole **65** is then reduced into amine **66**, using LiAlH_4_, which is in turn protected as *N*-Boc derivative **67** during the successive oxidation of the furan ring to yield acid **68**. Coupling between amine **69** and amide **70** is performed using bromotris-pyrrolidino-phosphonium hexafluorophosphate (PyBrOP) as an activating agent. The formation of amine **71** occurs after the treatment of **70** with thionyl chloride, and then cyclopropanemethylamine. ***Berotralstat*** was finally obtained as a single enantiomer after acidic Boc-deprotection and chiral SFC resolution.

### 4.2. Cedazuridine

***Cedazuridine***, in combination with *decitabine*, is sold under the brand name Inqovi and was developed by Otsuka Pharma. It was approved in July 2020 for the treatment of myelodysplastic syndromes (MDS) and chronic myelomonocytic leukemia (CMML), reducing the risk of progression of secondary acute myeloid leukemia (sAML) [52].

***Cedazuridine*** is a cytidine deaminase inhibitor that is able to improve the oral bioavailability of decitabine, avoiding its degradation in the gastrointestinal tract. The presence of two fluorine atoms at the ribose ring increase the level of metabolic stability under acidic conditions, improving the pharmacokinetic profile via unfluorinated analogs, retaining the same binding mode of unfluorinated tetrahydrouridines [53]. The synthesis of ***Cedazuridine*** is performed in two steps, starting with the analogue, ***Gemcitabine*** (Scheme 10). The Rh/C catalytic hydrogenation of ***Gemcitabine*** produces compound **76**, which is reduced into a mixture of isomers containing ***Cedazurine*** and its epimer as major products using NaBH_4_. The difluorotetrahydrofuran ring of ***Gemcitabine*** is synthesized via a Reformatzky reaction of fluorinated bromoacetate **72** with *D*-glyceraldehyde acetonide **73** to furnish **74** as a mixture of *anti/syn* diasteroisomers at a 3:1 ratio. The hydrolysis/lactonization of **74** into **75** is performed with Dowex 50 resin. Lactone **75** is then converted into ***Gemcitabine*** in four steps [54].

### 4.3. Pralsetinib

***Pralsetinib*** is sold under the brand name Gavreto and was developed by Blueprint Medicines [55]. It was approved in September 2020 for the treatment of metastatic fusion-positive non-small-cell lung cancer [56]. ***Pralsetinib*** is REarranged during transfection (RET) inhibitor and it is the first-in-class specific RET inhibitor with more selectivity than other kinases have. The presence of the 4-fluoropyrazolo group allows a different binding mode on the BP-II pocket, which is crucial for high-affinity binding and to avoid resistance from gatekeeper mutations [56]. 

4-Fluoropyrazole **77** gives nucleophilic displacement of bromopyridine **78** under basic conditions, yielding pyrazolylpyridine **79**. The stereoselective reductive amination to hydrochloride **82** is accomplished by means of the condensation of the acyl group of **79** with chiral sulfinamide **80**, followed by the reduction with L-Selectride and acidic hydrolysis of sulfinamide **81**. The latter fluorinated building block is coupled with acid **83** (as mixture of diastereoisomers) using PyBop as activating agents. A final chiral SFC resolution produces ***Pralsetinib*** as a single enantiomer. The method for the patented synthesis of ***Pralsetinib*** is described in Scheme 11 [57].

### 4.4. Selumetinib

***Selumetinib*** is sold under the brand name Koselugo and was developed by AstraZeneca. It was approved in April 2020 for the treatment of neurofibromatosis type 1 (NF1) [58]. ***Selumetinib***, characterized by the presence of a 4-fluorobenzimidazole core, is a mitogen-activated protein kinase (MEK) inhibitor that is able to target the Raf-MEK-ERK signaling pathway. The synthesis of ***Selumetinib*** is described in Scheme 12 [59].

Trifluorobenzoic acid **84** was employed as starting fluorinated building block for the initial construction of the fluorobenzimidazole ring. The nitration of **84** into **85** and the nucleophilic aromatic displacement of fluoride in nitro-activated derivative **85** with ammonia led to the acquisition of compound **86**. Treatment with trimethylsilyldiazomethane (TMS-CHN_2_) converts acid **86** into methyl ester **87**, which, in turn, reacts with aniline in xylene at 125 °C, causing the nucleophilic displacement of a second fluorine atom into **88**. The latter nitro derivative is reduced to *o*-diamminobenzene **89** via iron and ammonium chloride. The benzimidazole ring formation on **90** is performed with formamidine acetate (FAA) in EtOH at 80 °C. The halogenation of the anilino portion of **90** in two consecutive steps produces **91**, with the NBS-mediated introduction of bromine in the *para* position, and then, **92**, with *ortho*-chlorination performed with NCS. The methylation of **92** with methyliodide, employing K_2_CO_3_ in DMF, occurs at N-1, yielding regio-isomer **93**. Basic hydrolysis into acid **94** is followed by coupling with *O*-(2-(vinyloxy)ethyl)hydroxylamine in the presence of EDC and HOBt. Hydroxamic acid derivative **95** is finally deprotected via the acidic hydrolysis of the vinyl ether portions, producing ***Selumetinib***. 

### 4.5. Tauvid 

***Tauvid***, also named Flortaucipir F18, was developed by Eli Lilly and was approved in May 2020 as a positron emission tomography (PET) imaging probe for Alzheimer’s disease (AD) [60].

***Tauvid*** is the first approved tracer able to bind tau protein [61]. The [^18^F]fluoropyridine lateral ring is the radioactive portion of this probe. Two developed synthetic procedures are described in Scheme 13. 

The first synthetic approach is based on the nucleophilic displacement of the nitro group of precursor **96** with [^18^F]fluoride and using Kryptofix 222 (K_222_) as a phase transfer catalyst (PTC) [61]. This approach suffers from some drawbacks related to trace purity; therefore, a different synthetic approach was developed starting with N-Boc protected cation **97**, which undergoes nucleophilic displacement to produce radioactive fluorine, followed by acidic Boc removal [62]. This synthetic sequence allows the obtainment of ***Tauvid*** in higher yields and purity. Interestingly, this synthesis represents the only example of late-stage fluorination among all the molecules considered in this review. Obviously, the short half-life of ^18^F forces researchers to follow this peculiar synthetic approach.

## 5. FDA-Approved Drugs in 2019

**Scheme 14:**
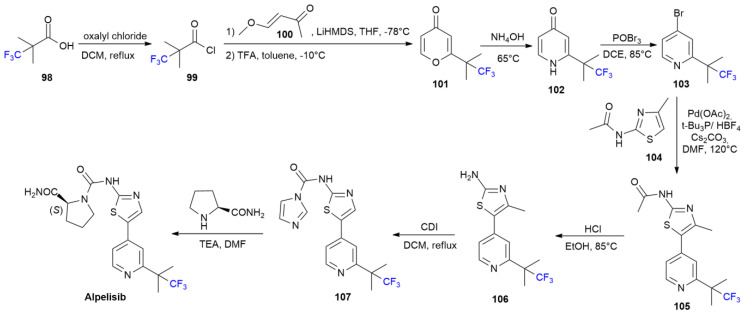
Synthesis of Apelisib.

**Scheme 15:**
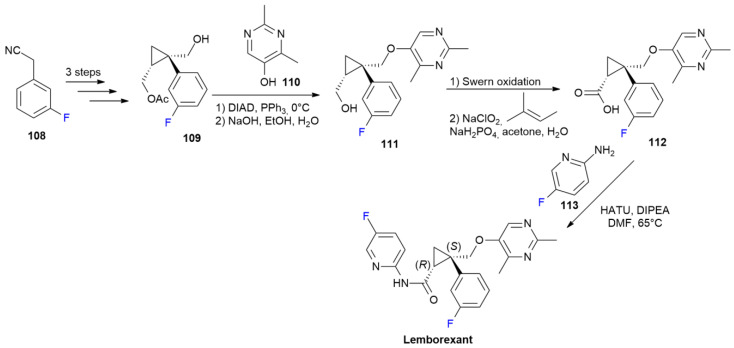
Synthesis of Lemborexant.

**Scheme 16:**
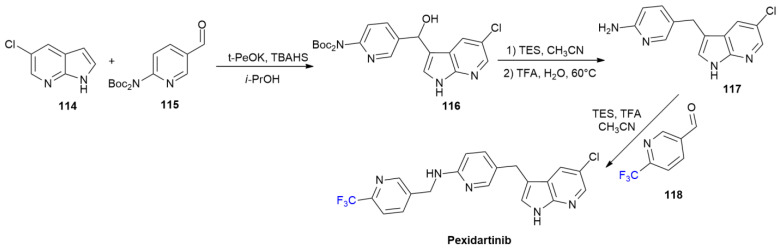
Synthesis of Pexidartinib.

**Scheme 17:**
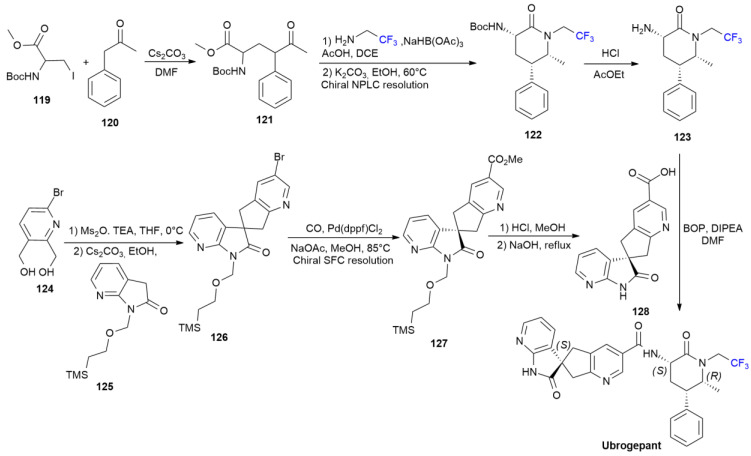
Synthesis of Ubrogepant.

In 2019, the FDA approved 48 new molecular entities, including 33 small molecules and 3 diagnostic agents [63]. Among the small molecules, 28 out of 33 contain at least one heterocyclic ring, and 11 out of 33 contain at least one fluorine atom [64,65]. In the following paragraph, four heterocyclic compounds bearing a fluorinated moiety directly linked to the ring are reported (Scheme 14, Scheme 15, Scheme 16 and Scheme 17). 

### 5.1. Alpelisib

***Alpelisib*** is sold under the brand name Rinvoq and was developed by Novartis. It was approved in May 2019 for the treatment of advanced or metastatic breast cancer [66]. ***Alpelisib*** is a phosphatidylinositol 3-kinase (PI3K) inhibitor possessing a trifluoro-*t*-butyl group at position two of the pyridine ring [67]. The presence of the fluorinated moiety induces a higher metabolic stability and excellent oral bioavailability. 

Furthermore, the fluorinated group is responsible for high affinity toward the PI3K binding site due to the hydrogen bond with K802, as revealed by X-ray data [68]. ***Alpelisib*** synthesis is performed using different approaches [69,70]. One method is shown in Scheme 14 [68].

Fluorinated acid **98** is first converted into the corresponding chloride, **99**, via oxalyl chloride under reflux. The acylation of the methyl group of enone **100** is performed at −78 °C using LiHMDS as a strong base. The intermediate diketone is directly cyclized to pyran-4-one **101** after a treatment with trifluoroacetic acid (TFA). The reaction of **101** with ammonium hydroxide produces fluorinated pyridin-4-one **102** via an ANRORC-like reaction. The treatment with POBr_3_ yields bromopyridine **103**, which is, in turn, coupled with acetylaminothiazole **104** using Pd(OAc)_2_ in a CH activation process. The resulting coupled product, **105**, is hydrolyzed using HCl into corresponding amine **106**. The treatment with carbonyldiimidazole (CDI) leads to intermediate **107**, which is then converted into ***Alpelisib*** after a treatment with *S*-prolinamide.

### 5.2. Lemborexant

***Lemborexant*** is sold under the brand name Dayvigo and was developed by Purdue Pharma L. ***Lemborexant*** is a dual orexin receptor antagonist, with fluorine in the position five of the pyridine moiety [71]. Another fluorine is also present at position three of the central phenyl ring. It was approved in December 2019 for the treatment of insomnia [72]. 

The presence of each fluorine is crucial to achieve high in vitro binding affinity, good solubility and a good pharmacological profile, as evidenced during the discovery process with the screening of different fluorination patterns [73].

The synthesis of ***Lemborexant*** starts with 3-fluorobenzyl cyanide **108**, which is converted into chiral cyclopropane derivative **109** in three steps (Scheme 15). Dimethylpyrimidine derivative **110** is used in a reaction under Mitsunobu conditions to obtain ether **111**. The conversion of the primary alcoholic function of **111** into corresponding carboxylic acid **112** is performed in two steps with Swern oxidation, followed by Pinnick oxidation. The final production of ***Lemborexant*** requires the coupling of acid **112** with 2-amino-5-fluoropyridine **113** using HATU as an activating agent.

### 5.3. Pexidartinib

***Pexidartinib*** is sold under the brand name Turalio and was developed by Daiichi Sankyo Inc. It was approved in August 2019 for the treatment of symptomatic tenosynovial giant cell tumor (TGCT) [74]. ***Pexidartinib*** is a tyrosine kinase inhibitor with selective efficacy for colony-stimulating factor (CSF) receptor; thus, it hampers the binding of CSF1 to CSF-receptor 1 (CSF1R).

Three steps in the synthesis of ***Pexidartinib*** at the kilogram scale are shown in Scheme 16 [75]. The base-mediated reaction of azaindole **114** at position 3, over aldehyde **115**, in the presence of tetrabutylammonium hydrogen sulphate (TBAHS) yields compound **116**. The dehydroxylation of **116** with triethylsilane (TES), followed by the Boc-deprotection of the 2-aminopyiridino moiety with TFA, produce compound **117**. ***Pexidartinib*** is obtained by means of the reductive amination of **117** with 6-(trifluoromethyl)nicotinaldehyde **118** using TES as a reducing agent.

### 5.4. Ubrogepant

***Ubrogepant*** is sold under the brand name Ubrelvy and was developed by Allergan. It was approved in December 2019 for the treatment of migraines with or without an aura in adults [76]. ***Ubrogepant*** is an effective calcitonin gene-related peptide (CGRP) receptor antagonist, bearing a chiral *N*-trifluoroethylpiperidinone ring, but its mechanism of action is still unknown. The synthesis of ***Ubrogepant*** was patented in 2012 and is reported in Scheme 17 [77]. The synthesis of fluorinated chiral amine **123**, starting with phenylacetone **120**, which alkylates with iodide **119** in the presence of Cs_2_CO_3_ as a base, produces derivative **121** in three steps. The reductive amination of the latter substance using trifluoroethylamine in the presence of sodium triacetoxyborohydride, leads to pyridinone **122** as a single enantiomer, after chiral resolution via normal-phase liquid chromatography (NPLC).

The treatment with HCl deprotects the N-Boc group, yielding **123**. The coupling of amine **123** with acidic spyro-subunit **128** (prepared as outlined in Scheme 17) using BOP as an activating agent yields ***Ubrogepant*** as a single enantiomer via SFC.

## 6. FDA-Approved Drugs in 2018

**Scheme 18:**
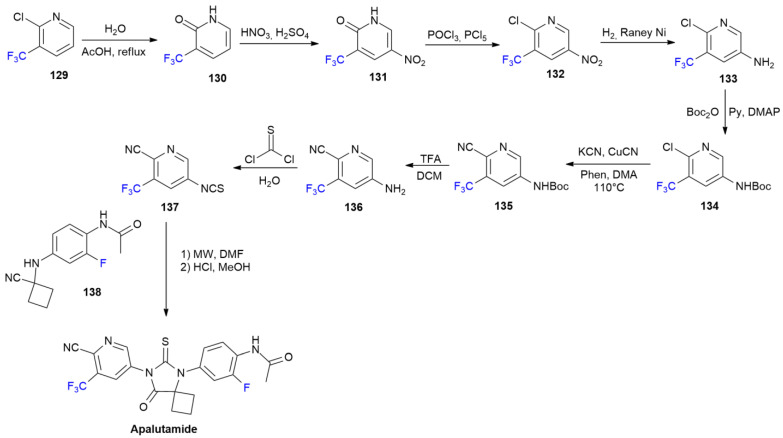
Synthesis of Aputalamide.

**Scheme 19:**
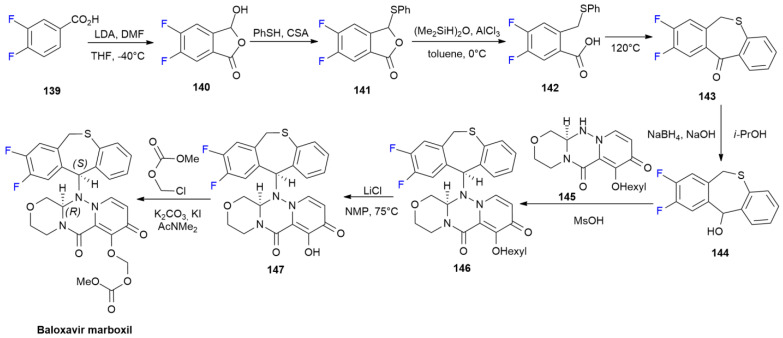
Synthesis of Baloxavir marboxil.

**Scheme 20:**
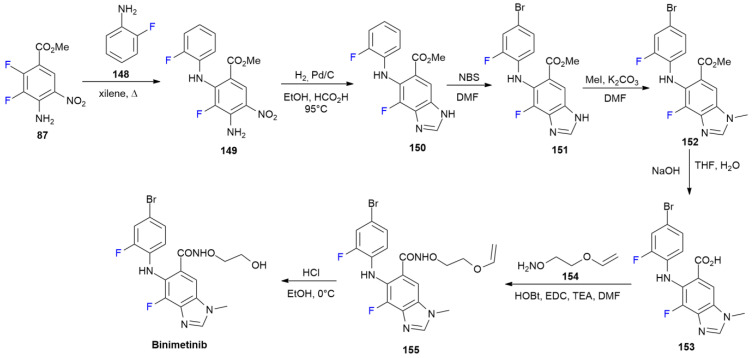
Synthesis of Binimetinib.

**Scheme 21:**
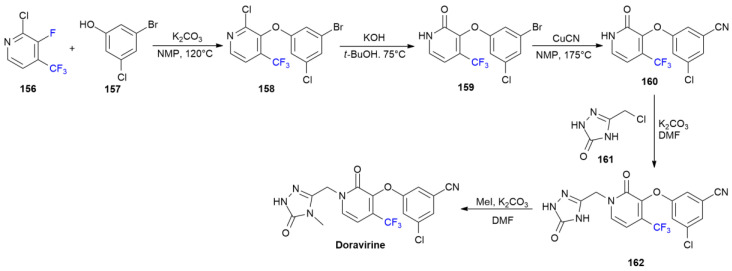
Synthesis of Doravirine.

**Scheme 22:**
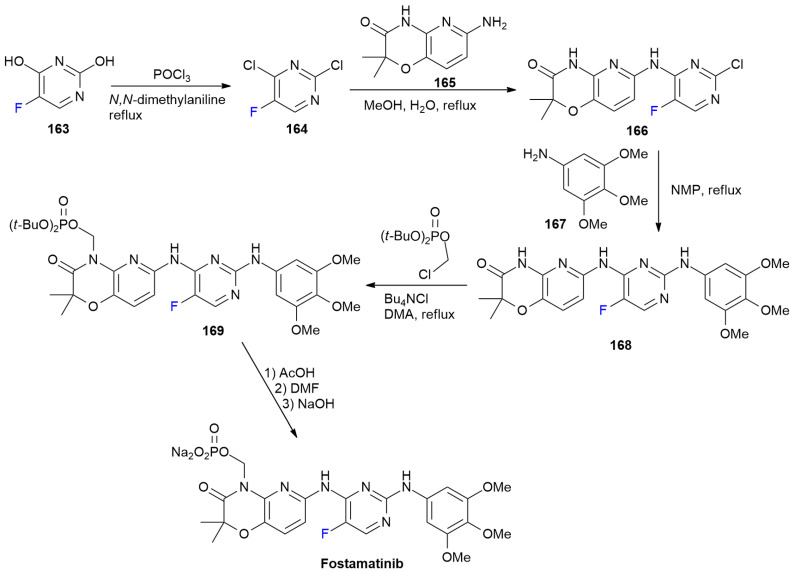
Synthesis of Fostamatinib.

**Scheme 23:**
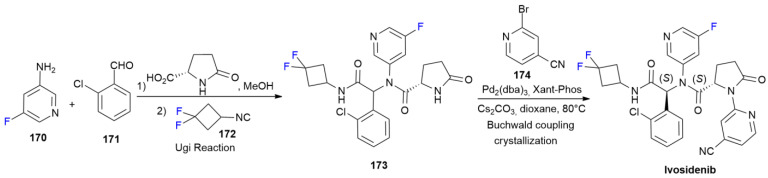
Synthesis of Ivosidenib.

**Scheme 24:**
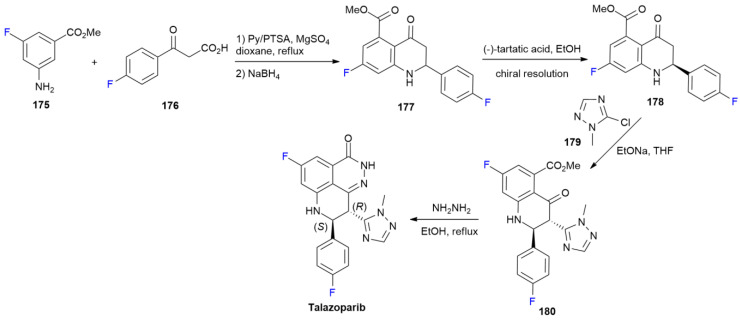
Synthesis of Talazoparib.

**Scheme 25:**
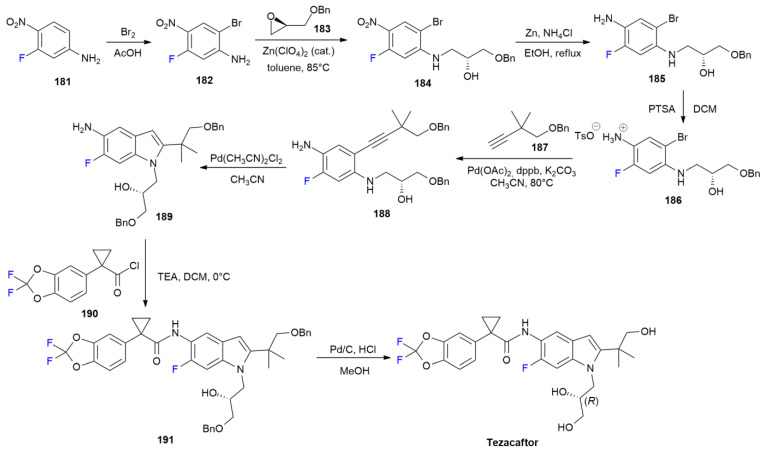
Synthesis of Tezacaftor.

In 2018, the FDA approved a collection of 59 new molecular entities, including 39 small molecules [78]. Thirty-two out of thirty-eight molecules contain at least one heterocyclic ring, and seventeen out of thirty-eight molecules contain at least one fluorine atom [16]. In the following paragraphs, eight heterocyclic compounds bearing a fluorinated moiety directly linked to the ring are reported (Scheme 18, Scheme 19, Scheme 20, Scheme 21, Scheme 22, Scheme 23, Scheme 24 and Scheme 25). 

### 6.1. Apalutamide 

***Apalutamide*** is sold under the brand name Erleada and was discovered by employees of the University of California and developed by Janssen [79]. It was approved in February 2018 for the treatment of prostate cancer (PC) [80]. ***Apalutamide*** is a non-steroidal oral androgen receptor inhibitor, presenting a trifluoromethylpyridine moiety linked to the central thiohydantoin core [81]. One of the initial patented ***Apalutamide*** synthesis procedures is reported below (Scheme 18) [82].

Chloro-trifluoromethylpyridine **129** is treated with water to induce the nucleophilic displacement of chlorine to obtain pyrimidone **130**. This compound can be easily nitrated into **131**, and again, converted into corresponding chloropyridine **132** via a treatment with PCl_5_/POCl_3_. The hydrogenation on Raney Ni into amine **133** is then followed by N-Boc protection with Boc-anhydride, yielding **134**. A Sandmeyer reaction causes cyanation to produce derivative **135**, which is subsequently deprotected into aminopyrimidine **136** using TFA. Isothiocyanate **137** is then obtained via the treatment the **136** using thiophosgene. ***Apalutamide*** is finally obtained from the construction of the thiohydantoin ring via the reaction of **137** with isocyanide **138** under microwave irradiation. The synthesis of crystalline forms of ***Apalutamide*** has recently been reviewed [83].

### 6.2. Baloxavir Marboxil

***Baloxavir marboxil*** is sold under the brand name Xofluza and was developed by Shionogi [84]. It was approved in October 2018 for the treatment of acute uncomplicated influenza types A and B [85].

***Baloxavir marboxil*** is a cap-dependent endonuclease inhibitor characterized by the presence of two fluorine atoms on the 6,11-dihydrodibenzo[b,e]thiepine ring [86]. The patented synthesis of ***Baloxavir marboxil*** is depicted in Scheme 19 [87]. 

Difluorobenzoic acid **139** is initially formylated via LDA/DMF at low temperature, yielding cyclization product **140**. A reaction with thiophenol under acidic conditions produces phenylthiolphthalide **141**. The reductive breaking of the C-O bond mediated by the action 1,1,3,3-Tetramethyldisiloxane yields thioether **142**, which undergoes intramolecular cyclization by heating at 120 °C. The obtained ketone **143** is reduced into alcohol **144** using NaBH_4_, and then coupled with chiral compound **145** under acidic conditions to produce compound **146**. The deprotection of hexyl ether into **147**, and a final reaction with chloromethyl methyl carbonate allows the obtainment of ***Baloxavir marboxil***.

### 6.3. Binimetinib

***Binimetinib*** is sold under the brand name Mektovi and was developed by Array Biopharma. It was approved in June 2018 for the treatment of metastatic BRAF V600E/K-positive advanced melanoma in association with Encorafenib [88]. 

***Binimetinib*** is a potent, selective, non-ATP competitive allosteric inhibitor of MEK1 and MEK2, with a fluorobenzimidazole moiety similar to that of ***Selumetinib*** (Scheme 12) [89].

As for the analogue, ***Selumetinib***, the synthetic route of ***Binimetinib*** is based on ester **87** (Scheme 20) [90]. The nucleophilic displacement of 2-fluoroaniline **148** to obtain derivative **149** is followed by hydrogenation in the presence of formic acid to directly yield benzimidazole **150**. NBS-mediated bromination and methylation at N-1 give compounds **151** and **152**, respectively. Ester **117** is, therefore, hydrolyzed using NaOH, and the resulting acid, **153**, after activation using EDC/HOBt is converted into hydroxamic acid **155** upon a reaction with O-alkyl hydroxylamine **154**. ***Binimetinib*** is finally obtained by the acidic hydrolysis of the vinyl ether group of **155**.

### 6.4. Doravirine

***Doravirine*** is sold under the brand name Pifeltro and was developed by Merck. It was approved in August 2018 for the treatment of human immunodeficiency virus 1 (HIV-1) infections [91]. ***Doravirine*** is a non-nucleoside reverse transcriptase inhibitor, presenting improved ADME properties due the presence of a CF_3_-substituted pyridone central ring. In fact, the presence of this strong electron-withdrawing group is correlated with a longer elimination half-life in rats and dogs compared to that of unfluorinated analogs [92]. The patented method of the synthesis of ***Doravirine*** is shown in Scheme 21 [93].

Fluorinated building block 2-chloro-3-fluoro-4-(trifluoromethyl)pyridine **156** is first used in a reaction with phenol **157** using K_2_CO_3_ as a base to induce fluoride displacement and obtain ether **158**. The hydrolysis of chloropyridine **158** into pyridinone **159** is then performed via a treatment with KOH. The cyanation of the C-Br bond of the phenyl portion with CuCN yields substrate **160**, which reacts under basic condition with chloromethyltriazole **161** to produce pyridone *N*-alkylation product **162**. ***Doravirine*** is finally obtained by the regio-selective methylation of the 1,2,4-triazole ring at N-4 with methyliodide in DMF in the presence of K_2_CO_3_ as a base. Other synthetic approaches have recently been reviewed [16].

### 6.5. Fostamatinib

***Fostamatinib*** is sold under the brand name Tavalisse and was developed by Rigel Pharmaceuticals. It was approved in April 2018 for the treatment of thrombocytopenia in adults with persistent or chronic immune thrombocytopenia (ITP) [94]. 

***Fostamatinib*** is a potent spleen tyrosine kinase (Syk) inhibitor, bearing a 5-fluoropyrimidine ring, and it is used to improve membranes’ permeability [95]. Actually, ***Fostamatinib*** is a pro-drug of compound **168**, and its synthesis is described in Scheme 22 [96]. 

5-Fluoropyrimidine-2,4-diol **163** is converted into dichloro-derivative **164** after a treatment with POCl_3_. 

Two subsequent chloride displacements with different amines then occur. The first one at C-4 with amino-pyridoxazinone **165** yields **166**; the second one at C-2 with 3,4,5-trimethoxyaniline **167** produces compound **168**. As mentioned above, this compound is converted into the pro-drug ***Fostamatinib*** via a treatment with di-tert-butyl(chloromethyl)phosphate to produce ester **169**, which is then hydrolyzed and converted into target phosphate disodium salt.

### 6.6. Ivosidenib 

***Ivosidenib*** is sold under the brand name Tibsovo and was developed by Servier. It was approved in July 2018 for the treatment of relapsed or refractory acute myeloid leukemia [97]. ***Ivosidenib*** is an inhibitor of mutated cytosolic isocitrate dehydrogenase 1 (IDH1); thus, it lowers the level of oncometabolite *D*-2-hydroxyglutarate (2-HG) [98]. Fluorine at the position five of the pyridine ring is crucial in order to ensure a high level of potency and metabolic stability [98]. The synthesis of ***Ivosidenib*** at the multi-gram scale is outlined in Scheme 23 [99]. The synthetic process is based on a multi-component Ugi reaction of 3-amino-5-fluoropyridine **170** with 2-chlorobenzaldehyde **171**, followed by *L*-pyroglutamic acid and isocyanide **172**, to produce peptide derivative **173**. N-H coupling with bromopyridine **174** under Buchwald conditions produces ***Ivosidenib*** as a single enantiomer after crystallization.

### 6.7. Talazoparib

***Talazoparib*** is sold under the brand name Talzenna and was developed by Pfizer [100]. It was approved in October 2018 for the treatment of locally advanced or metastatic breast cancer patients with a germline BRCA mutation [101]. ***Talazoparib***, a fluorine-containing tetrahydropyridophthlazinones is active as a poly(ADP-ribose) polymerase (PARP) inhibitor [101]. To achieve inhibitory activity and metabolic stability, as well as to increase the number of interactions at the binding site via H-bonding, 5-fluoro substitution and the 4-fluorophenyl groups are crucial [101]. The synthetic method for the preparation of ***Talazoparib*** at the 30 g scale is described in Scheme 24 [102]. 

Fluorinated dihydroquinolinone **177** is constructed with a two steps acylation/reductive amination of fluoroaniline **175** with β-ketoacid **176**. The following chiral resolution with (-)-tartaric acid is crucial to obtain the desired enantiomer, **178**. The base-induced reaction between **178** and 5-chloro-1-methyl-1,2,4-triazole **179** leads to the desired trans stereoisomer, **180**, which is finally converted into ***Talazoparib*** via a reaction with hydrazine in EtOH under reflux.

### 6.8. Tezacaftor

***Tezacaftor*** is sold under the brand name Symdeko, as a co-formulation with ivacaftor and was developed by Vertex Pharms Inc. It was approved in February 2018 for the treatment of cystic fibrosis [103]. ***Tezacaftor*** improves the processing and trafficking of cystic fibrosis transmembrane conductance regulator (CFTR) in vitro and improves CFTR’s function alone and in combination with other drugs [104]. 

The synthesis of ***Tezacaftor*** has been recently reviewed [105], the second generation process is described in Scheme 25 [106]. 

The scheme is based on the initial formation of 6-fluoroindole’s nucleus using 3-fluoro-4-nitroaniline **181**. Bromination with elemental bromine in acetic acid yields compound **182**, and the nucleophilic ring opening by the anilino moiety on chiral epoxide **183** produces compound **184**. Nitro-group reduction with Zinc and salt formation with PTSA from amine **185**,produce tosylate **186**. The latter one is coupled with terminal alkyne **187** under Sonogashira conditions to produce compound **188**. Indole ring formation is achieved via a Pd-catalyzed reaction using Pd(CH_3_CN)_2_Cl_2_. 6-Fluoroindole **189**, obtained as a single enantiomer, is then used in a reaction with chloride **190** to yield Bn-protected derivative **191**, which is finally converted into ***Tezacaftor*** by means of hydrogenation over Pd/C.

## 7. FDA-Approved Drugs in 2017

**Scheme 26:**
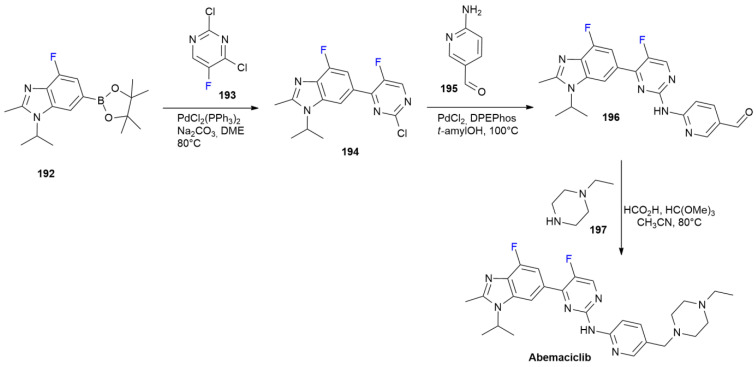
Synthesis of Abemaciclib.

**Scheme 27:**
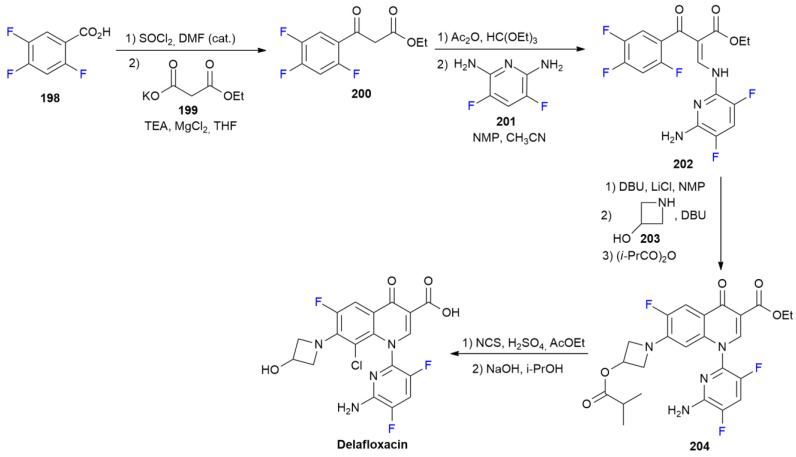
Synthesis of Delafloxacin.

**Scheme 28:**
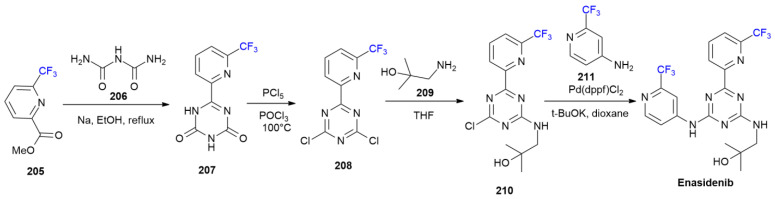
Synthesis of Enasidenib.

**Scheme 29:**
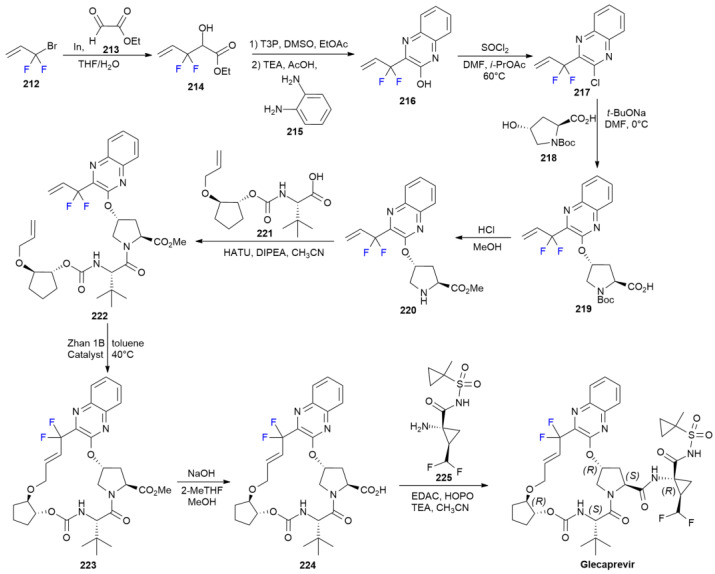
Synthesis of Glecaprevir.

**Scheme 30:**
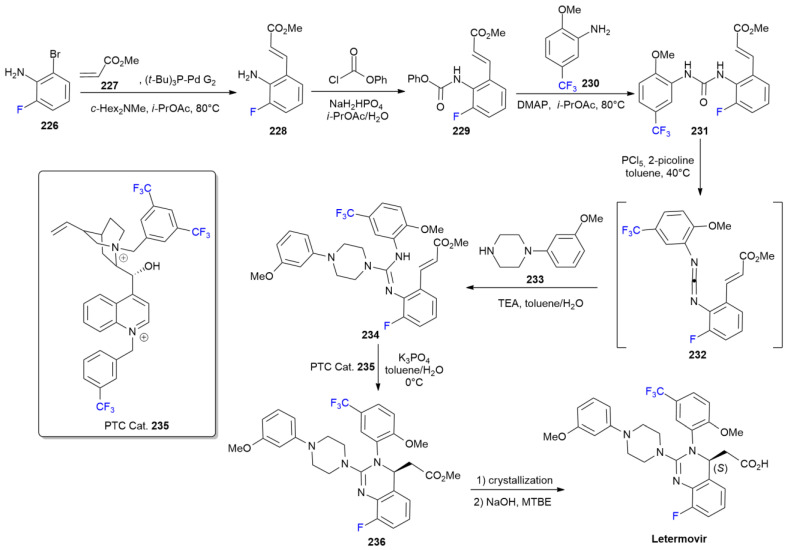
Synthesis of Letermovir.

**Scheme 31:**
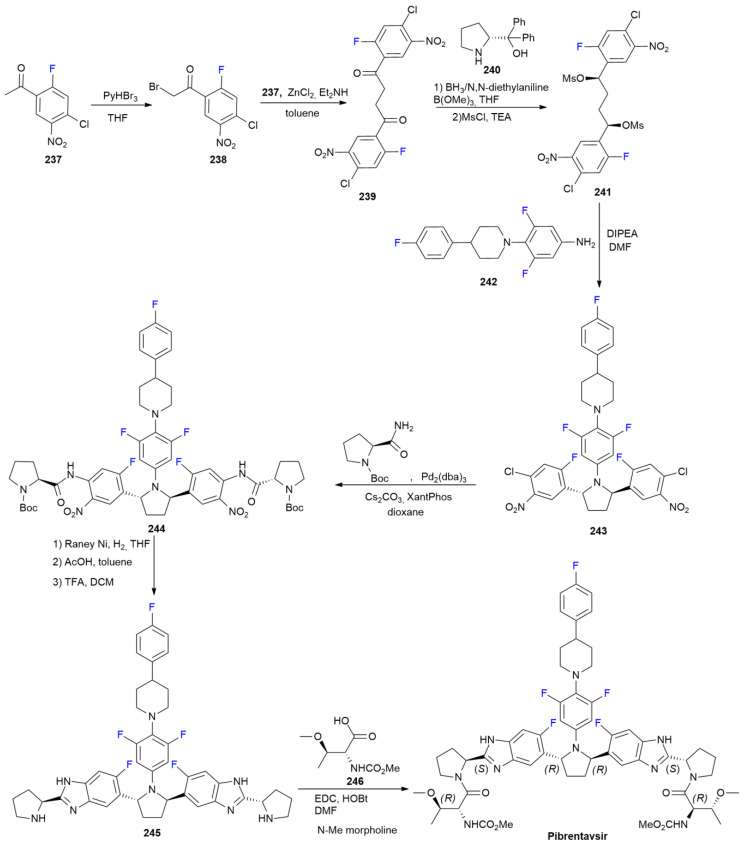
Synthesis of Pibrentasvir.

**Scheme 32:**
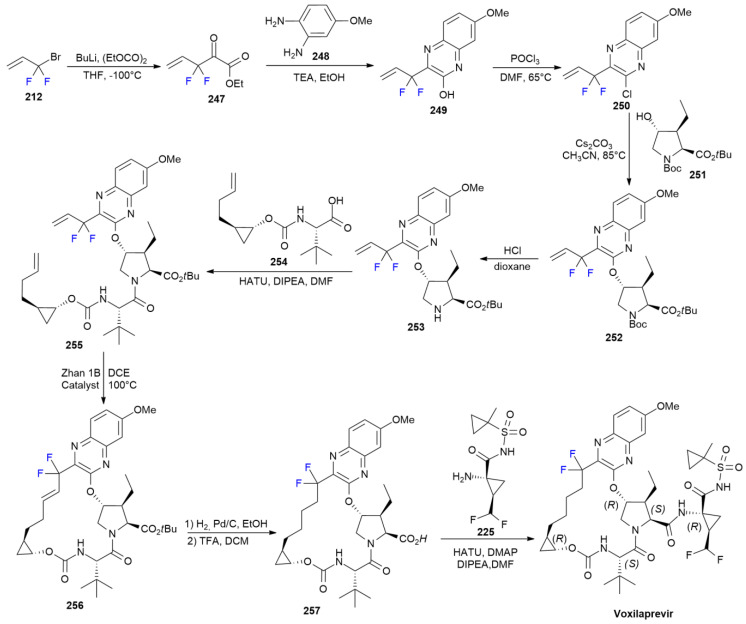
Synthesis of Voxilaprevir.

In 2017, the FDA approved 46 new drugs, including 34 small molecules [107,108]. Thirty-one out of thirty-four molecules contain at least one heterocyclic ring, and ten out of thirty-four molecules contain at least one fluorine atom. In the following paragraphs, seven heterocyclic compounds bearing a fluorinated moiety directly linked to the ring are reported (Scheme 26, Scheme 27, Scheme 28, Scheme 29, Scheme 30, Scheme 31 and Scheme 32).

### 7.1. Abemaciclib 

***Abemaciclib*** is sold under the brand name Verzenio and was developed by Eli Lilly. It was approved in September 2017 for the treatment of advanced or metastatic breast cancers. ***Abemaciclib*** is a cyclin-dependent kinase (CDK) inhibitor that is selective for isoforms CDK4 and CDK6 [109]. ***Abemaciclib*** contains two fluorinated heterocycles, namely 4-fluorobenzimidazole and 5-fluoropyrimidine, which are directly linked to form a 6-(pyrimidin-4-yl)-benzimidazole core (Scheme 26). The synthesis described by Frederick et al. starts with the formation of this bond via a Suzuki reaction between fluorobenzimidazolyl pinacol boronate **192** and 2,4-dichloro-5-fluoropyrimidine **193** [110]. The reaction occurs selectively with the displacement of chlorine at position 4, producing **194**, while less-reactive chlorine at position 2 is then used in a reaction with aminopyridine **195** under Buchwald–Hartwig conditions to yield intermediate **196**. The latter substance is converted into ***Abemaciclib*** through reductive amination with ethylpyperazine **197** via a Leuckart–Wallach reaction, with trimethyl orthoformate as a dehydrating agent. A further improvement has introduced a more convergent scheme, which involves the performance of flow synthesis [111].

### 7.2. Delafloxacin

***Delafloxacin*** is sold under the brand name Baxdela and was developed by Melinta. It was approved in June 2017 for the treatment of acute bacterial skin and skin structure infections [112]. Such as other members of the fluoroquinolone family, it is a DNA gyrase topoisomerase IV inhibitor that is active against Gram-positive bacteria, including methicillin-resistant *Staphylococcus Aureus* (MRSA), and Gram-negative organisms, such as *Escherichia Coli* and *Pseudomonas Aeruginosa* [113].

Additionally, some quinolone-resistant strains are susceptible to ***Delafloxacin***. The synthetic process is in line with the classical fluoroquinolone method (Scheme 27) [114]. 

Trifluorobenzoic acid **198** is initially converted into the corresponding chloride with thionylchloride, and then into β-ketoester **200** via a treatment with potassium monoethylmalonate **199**. Compound **200** is then converted into an intermediate vinylether, which is directly transformed into enamine **202** after the reaction with 2,6-diamino-3,5-difluoropyridine **201**. The cyclization of compound **202** into the corresponding quinolone by the nucleophilic displacement of *ortho* fluorine is induced by the addition of DBU. The second aromatic nucleophilic substitution, involving fluorine at position seven, is performed with 3-hydroxyazetidine **203**. Compound **204** is then obtained via the protection of an hydroxyl group as an ester to avoid competitive oxidation in the following chlorination step. Chlorination at position eight of the quinolone ring is selectively performed using NCS as a chlorinating agent in an acidic environment. Finally, ***Delafloxacin*** is obtained after the deprotection of the hydroxyazetine portion by means of ester hydrolysis with NaOH.

### 7.3. Enasidenib

***Enasidenib*** is sold under the brand name Idhifa and was developed by Celgene. It was approved in August 2017 for the treatment of relapsed or refractory acute myeloid leukemia in patients with specific mutations of the isocitrate dehydrogenase 2 (IDH2) gene [115]. ***Enasidenib*** is a first-in-class small-molecule inhibitor of the IDH2-mutant enzyme with oral bioavailability [116]. This drug contains two trifluoromethylpyridine rings, as demonstrated by ab initio calculations with X-ray data; one trifluoromethyl group is important for the CF_3_···O tetrel bond with Asp312 [117]. The same CF_3_-group is also responsible for C-H···F bonding with Asp312 and N-H···F bonding with Gln316. The synthesis of ***Enasidenib*** was patented in 2013 (Scheme 28) [118].

Trifluoromethylpycolinate **205** is condensed using biuret **206** in refluxing EtOH in the presence of sodium metal to produce 1,3,5-triazin-2,4-dione **207**. Chlorination with PCl_5_ in POCl_3_ produces dichlorotriazine **208**. The nucleophilic displacement of aminoalcohol **209** produces compound **210**. The Buchwald–Hartwig Pd-catalyzed N-arylation of 4-amino-2-(trifluoromethyl)pyridine **211** with chloride **210** forms ***Enasidenib***.

### 7.4. Glecaprevir

***Glecaprevir*** is sold under the brand name Mavyret, as a co-formulation with ***Pibrentasvir*** (see Section 7.6), and was developed by AbbVie Inc. It was approved in August 2017 for the treatment of chronic hepatitis C virus (HCV) in adults [119].

In 2019, the FDA expanded the use to children. ***Glecaprevir*** is a non-structural (NS) protein 3/4A protease inhibitor, presenting a macrocyclic ring with a difluoromethylene moiety directly linked to a quinoxaline ring [120]. The enabling synthesis of **Glecaprevir** to produce the quantity needed for Phase I clinical trials is based on ring-closing metathesis (RCM) for the production of the 18-membered macrocycle (Scheme 29) [121].

The synthetic route starts with the formation of fluorinated α-hydroxyester **214** via the Indium-mediated allylation of ethyl glyoxylate **213** starting with 3-bromo-3,3-difluoro-propene **212**. The Swern oxidation of propylphosphonic anhydride (T3P) into intermediate α-ketoester is followed by condensation with ortho-phenylenediamine **215** to produce *gem*-difluoro quinoxaline **216**. Chlorination with thionyl chloride produces derivative **217**, possessing a good leaving group for nucleophilic aromatic substitution with Boc-protected hydroxyproline **218**. Concurrent methyl ester formation and the removal of Boc protection via treating **219** with HCl in MeOH produces amine **220**, which is one of the two main building blocks for macrocycle formation. The second main component, acid **221**, is coupled, inducing amide bond formation when employing HATU as an activating agent. Diene **222** is then subjected to RCM using Zhan 1B catalyst after careful screening of the reaction conditions, thereby optimizing the formation of the desired *trans* macrocycle, **223**. ***Glecaprevir*** is obtained in two steps via the hydrolysis of **223** into acid **224** and its coupling with the fluorinated aminocyclopropane **225** side-chain, combining EDC and 2-hydroxypyridine-*N*-oxide (HOPO) as activating agents. Another approach based on ether-bond macrocyclization has also been developed for large-scale synthesis [122].

### 7.5. Letermovir

***Letermovir*** is sold under the brand name Prevymis and was developed by Merck & Co. It was approved in November 2017 for the treatment of infections caused by cytomegalovirus (CMV) after a bone marrow transplant [123]. ***Letermovir’s*** mode of action is different from that of other antiviral agents, which act on DNA polymerase; in fact, it interferes with the activity of terminase complex of the virus [124]. The asymmetric synthesis of ***Letermovir*** is performed in seven steps with a key part: PTC-mediated aza-Michael cyclization to obtain the chiral fluorinated dihydroquinazoline core (Scheme 30) [125]. The formation of aminocinnamate **228** is based on a Heck reaction between fluoroaniline **226** and methyl acrylate **227**. Carbamate **229** is then obtained treating **228** with phenyl chloroformate. Urea **231** is formed via a reaction with anisidine **230**. Compound **231** is dehydrated with PCl_5_ into carbodiimide **232** and directly converted into guanidine **234** via a treatment with piperazine **233**. Compound **234** is the key intermediate used for asymmetric cyclization into compound **236** using fluorinated cinchona-based derivative **235** as a PTC catalyst. Precursor **236** is converted into ***Letermovir*** via the hydrolysis of the methyl ester moiety. Other asymmetric approaches to ***Letermovir*** synthesis were developed later [126,127].

### 7.6. Pibrentasvir

***Pibrentasvir*** is sold under the brand name Mavyret, as a co-formulation with ***Glecaprevir*** (see Scheme 29), and was developed by AbbVie Inc. It was approved in August 2017 for the treatment of chronic hepatitis C virus (HCV) in adults [119]. ***Pibrentasvir*** is an NS5A inhibitor antiviral agent with two symmetric fluorobenzimidazole rings linked to a central *trans* pyrrolidine core [128]. The method for the patented synthesis of ***Pibrentasvir*** is shown in Scheme 31 [129].

Fluoro-acetophenone **237** was brominated using the methyl group, producing α-bromoketone **238**. The ZnCl_2_-mediated C-C coupling of **237** with **238** produces diketone **239**. Stereoselective reduction in the presence of prolinol-derived catalyst **240** yields an intermediate diol, which is directly converted into dimesylate **241**. Double nucleophilic displacement with aniline **242** produces trans pyrrolidine **243**, which is then treated with N-Boc prolinamide under Buchwald conditions to yield N-arylation at both rings in compound **244**. The latter substance is converted into bis-benzimidazole **245** via the hydrogenation of nitro groups, AcOH-mediated cyclization and TFA-induced deprotection. Diamine **245** is finally converted into ***Pibrentasvir*** via coupling with protected *O*-methyl-threonine **246** with EDC and HOBt as an activating agent.

### 7.7. Voxilaprevir

***Voxilaprevir*** is sold under the brand name Vosevi, as a co-formulation with ***sofosbuvir*** (see Section 8.2) and ***velpatasvir*** and was developed by Gilead. It was approved in July 2017 for the treatment of chronic hepatitis C virus (HCV) in adults [130]. ***Voxilaprevir*** is an NS protein 3/4A protease inhibitor, possessing an 18-membered *gem*-difluoro methylene quinoxaline portion similar to that of ***Glecaprevir*** (Scheme 29). Additionally, the synthetic pathways are quite similar (Scheme 32) [131]. 

The synthetic method starts with the formation of fluorinated α-ketoester **247** via the lithium exchange and allylation of diethyl oxalate, starting with 3-bromo-3,3-difluoro-propene **212**. Condensation with methoxy ortho-phenylenediamine **248** produces *gem*-difluoro quinoxaline **249**, which is chlorinated with POCl_3_ to produce derivative **250**, which possesses a good leaving group for nucleophilic aromatic substitution with Boc-protected ethyl hydroxyproline **251**. The Boc removal of **252** with HCl furnishes amine **253**, which is one of the two main building blocks for macrocycle formation. The second main component, acid **254**, is coupled to it by inducing amide bond formation and employing HATU as an activating agent. Diene **255** is then subjected to RCM using Zhan 1B catalyst, causing the formation of the desired *trans* macrocycle, 256. ***Glecaprevir*** is obtained in two steps by means of the hydrogenation of the double bond of **256** and *t*-Bu ester removal to produce acid **257** and its coupling with fluorinated aminocyclopropane **225** side-chain using HATU as an activating agent.

## 8. FDA-Approved Drugs in 2016

**Scheme 33:**
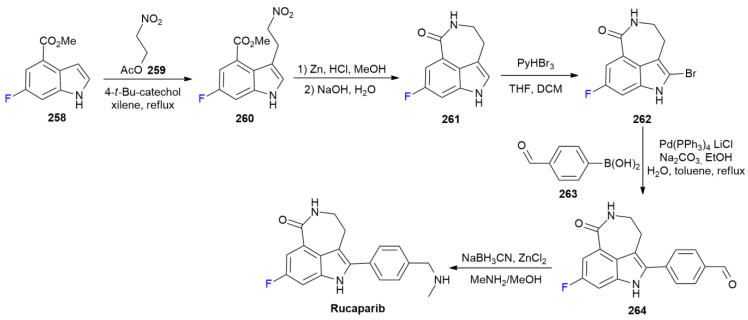
Synthesis of Rucaparib.

**Scheme 34:**
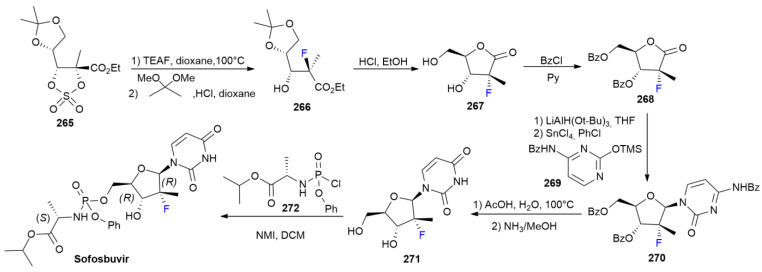
Synthesis of Sofosbuvir.

In 2016, the FDA approved 22 new drugs, including 13 small molecules [132]. Eleven out of thirteen molecules contain at least one heterocyclic ring, and four out of thirteen molecules have at least one fluorine atom. In the following paragraphs, two heterocyclic compounds bearing a fluorinated moiety directly linked to the ring are reported (Scheme 33 and Scheme 34). 

### 8.1. Rucaparib

***Rucaparib*** is sold under the brand name Rubraca and was developed by Clovis Oncology. It was approved in December 2016 for the treatment of ovarian cancer [133]. ***Rucaparib*** is the first-in-class inhibitor of DNA repair enzyme poly-ADP ribose polymerase-1 (PARP-1); notably, the presence of fluorine at the indole ring enhances the in vitro potency tenfold in comparison to that of the unfluorinated analogue [134]. ***Rucaparib*** synthesis is completed in five steps, starting with fluoroindole **258** (Scheme 33) [135]. The alkylation of **258** with nitroacetate **259** yields compound **260**, which is reduced using Zinc in an acid medium and directly cyclized under basic conditions into azepino-indole **261**. The bromuration of indole C-2 produces **262**, which undergoes to a Suzuki reaction with formyl boronic acid **263**. The obtained aldehyde **264** is converted into ***Rucaparib*** by means of reductive amination with NaBH_3_CN.

### 8.2. Sofosbuvir

***Sofosbuvir*** is sold under the brand name Epclusa, as a co-formulation with *velpatavsir*, and was developed by Gilead Science. It was approved in June 2016 for the treatment of six major forms of HCV [136]. ***Sofsbuvir*** has also been FDA-approved since 2014 for the treatment of HCV, alone or co-administered with other drugs [137]. ***Sofosbuvir*** acts as an HCV NS5B polymerase inhibitor and is administered as a prodrug [138]. The synthesis of ***Sofosbuvir*** was described by Bao et al. in 2010 (Scheme 34). ***Sofosbuvir*** was originally obtained from cytidine derivative **270** via the hydrolysis of the amino group and Bz removal, followed by the installation of a phosphoramidate side-chain on **272** in the presence of *N*-methylimidazole (NMI) [139]. Compound **270** has been previously synthesized, starting with chiral cyclic sulfate **265** [140]. Sulfate opening using fluoride is followed by a hydrolytic step, allowing the obtainment of fluorinated chiral compound **266**. Acetonide hydrolysis, performed with HCl in EtOH, straightforwardly produces lactone **267**, which is protected as it is Bz ester **268**, and then reduced and coupled with pyrimidine derivative **269** to produce compound **270**.

## 9. Conclusions

The use and the importance of fluorinated heterocyclic compounds is continuously increasing in the field of medicinal chemistry. Drug discovery is often linked to the presence of a fluorinated moiety that is able to improve the drug’s potency or the metabolic stability of different heterocycles. These features allow the constant increase in these molecular entities, among other new drugs and among blockbuster and best-selling compounds. The only restriction, up to now, could be due to the limitation of fluorinated building blocks for synthetic purposes. In fact, all the reported synthetic schemes are based on available fluorinated heterocyclic compounds or their precursors, while late fluorination strategies are limited to ^18^F probes due to the short half-life of this isotope. The installation of fluorinated moieties at the end of the synthesis could be a different approach that could characterize future research in this field.

## Data Availability

Not applicable.

## References

[1] Kerru N., Gummidi L., Maddila S., Gangu K.K., Jonnalagadda S.B. (2020). A Review on Recent Advances in Nitrogen-Containing Molecules and Their Biological Applications. Molecules.

[2] Martorana A., Giacalone V., Bonsignore R., Pace A., Gentile C., Pibiri I., Buscemi S., Lauria A., Piccionello P.A. (2016). Heterocyclic Scaffolds for the Treatment of Alzheimer’s Disease. Curr. Pharm. Des..

[3] Pathania S., Narang R.K., Rawal R.K. (2019). Role of sulphur-heterocycles in medicinal chemistry: An update. Eur. J. Med. Chem..

[4] Wetzel C., Lonneman M., Wu C. (2021). Polypharmacological drug actions of recently FDA approved antibiotics. Eur. J. Med. Chem..

[5] Heravi M.M., Zadsirjan V. (2020). Prescribed drugs containing nitrogen heterocycles: An overview. RSC Adv..

[6] Inoue M., Sumii Y., Shibata N. (2020). Contribution of Organofluorine Compounds to Pharmaceuticals. ACS Omega.

[7] Upadhyay C., Chaudhary M., De Oliveira R.N., Borbas A., Kempaiah P.S., Rathi B. (2020). Fluorinated scaffolds for antimalarial drug discovery. Expert Opin. Drug Discov..

[8] O’Hagan D. (2010). Fluorine in health care: Organofluorine containing blockbuster drugs. J. Fluor. Chem..

[9] Fried J., Sabo E.F. (1954). 9α-Fluoro Derivatives of Cortisone and Hydrocortisone. J. Am. Chem. Soc..

[10] Purser S., Moore P.R., Swallow S., Gouverneur V. (2008). Fluorine in medicinal chemistry. Chem. Soc. Rev..

[11] Meanwell N.A. (2018). Fluorine and Fluorinated Motifs in the Design and Application of Bioisosteres for Drug Design. J. Med. Chem..

[12] Johnson B.M., Shu Y.Z., Zhuo X., Meanwell N.A. (2020). Metabolic and Pharmaceutical Aspects of Fluorinated Compounds. J. Med. Chem..

[13] Morgenthaler M., Schweizer E., Hoffmann-Röder A., Benini F., Martin R.E., Jaeschke G., Wagner B., Fischer H., Bendels S., Zimmerli D. (2007). Predicting and Tuning Physicochemical Properties in Lead Optimization: Amine Basicities. ChemMedChem.

[14] Rowley M., Hallett D.J., Goodacre S., Moyes C., Crawforth J., Sparey T.J., Patel S., Marwood R., Patel S., Thomas S. (2001). 3-(4-Fluoropiperidin-3-yl)-2-phenylindoles as High Affinity, Selective, and Orally Bioavailable h5-HT2A Receptor Antagonists. J. Med. Chem..

[15] Shah P., Westwell A.D. (2007). The role of fluorine in medicinal chemistry. J. Enzym. Inhib. Med. Chem..

[16] Mei H., Han J., Fustero S., Medio-Simon M., Sedgwick D.M., Santi C., Ruzziconi R., Soloshonok V.A. (2019). Fluorine-Containing Drugs Approved by the FDA in 2018. Chem. A Eur. J..

[17] Knight J.C., Edwards P.G., Paisey S.J. (2011). Fluorinated contrast agents for magnetic resonance imaging; a review of recent developments. RSC Adv..

[18] Van der Born D., Pees A., Poot A.J., Orru R.V.A., Windhorst A.D., Vugts D.J. (2017). Fluorine-18 labelled building blocks for PET tracer synthesis. Chem. Soc. Rev..

[19] Das P., Delost M.D., Qureshi M.H., Smith D.T., Njardarson J.T. (2019). A Survey of the Structures of US FDA Approved Combination Drugs. J. Med. Chem..

[20] Ivasyshyn V., Smit H., Chiechi R.C. (2019). Synthesis of a Hominal Bis(difluoromethyl) Fragment. ACS Omega.

[21] Liang T., Neumann C.N., Ritter T. (2013). Introduction of Fluorine and Fluorine-Containing Functional Groups. Angew. Chem. Int. Ed..

[22] López S.E., Salazar J. (2013). Trifluoroacetic acid: Uses and recent applications in organic synthesis. J. Fluor. Chem..

[23] Shibata N., Matsnev A., Cahard D. (2010). Shelf-stable electrophilic trifluoromethylating reagents: A brief historical perspective. Beilstein J. Org. Chem..

[24] Eisenberger P., Gischig S., Togni A. (2006). Novel 10-I-3 Hypervalent Iodine-Based Compounds for Electrophilic Trifluoromethylation. Chem. A Eur. J..

[25] Magnier E., Blazejewski J.-C., Tordeux M., Wakselman C. (2006). Straightforward One-Pot Synthesis of Trifluoromethyl Sulfonium Salts. Angew. Chem. Int. Ed..

[26] FDA (2022). Novel Drug Approvals for 2022.

[27] De la Torre B.G., Albericio F. (2023). The Pharmaceutical Industry in 2022: An Analysis of FDA Drug Approvals from the Perspective of Molecules. Molecules.

[28] Paik J. (2022). Lenacapavir: First Approval. Drugs.

[29] Segal-Maurer S., DeJesus E., Stellbrink H.-J., Castagna A., Richmond G.J., Sinclair G.I., Siripassorn K., Ruane P.J., Berhe M., Wang H. (2022). Capsid Inhibition with Lenacapavir in Multidrug-Resistant HIV-1 Infection. N. Engl. J. Med..

[30] Graupe M., Henry S.J., Link J.O., Rowe C.W., Saito R.D., Schroeder S., Stefanidis D., Tse W.C., Zhang J.R. (2018). Therapeutic Compounds Useful for the Prophylactic or Treatment of an HIV Virus Infection. Patent WO.

[31] Hoy S.M. (2022). Oteseconazole: First Approval. Drugs.

[32] Sobel J.D., Nyirjesy P. (2021). Oteseconazole: An advance in treatment of recurrent vulvovaginal candidiasis. Future Microbiol..

[33] Hoekstra W.J., Schotzinger R.J., Rafferty S.W. (2011). Metalloenzyme Inhibitor Compounds. Patent WO.

[34] Benedetto Tiz D., Bagnoli L., Rosati O., Marini F., Sancineto L., Santi C. (2022). New Halogen-Containing Drugs Approved by FDA in 2021: An Overview on Their Syntheses and Pharmaceutical Use. Molecules.

[35] Deeks E.D. (2022). Atogepant: First Approval. Drugs.

[36] Hay D.L., Walker C.S., Harris P.W.R. (2022). Atogepant (Qulipta^®^) for migraine prevention. Trends Pharmacol. Sci..

[37] Belyk K.M., Cleator E., Kuo S., Maligres P.E., Xiang B., Yasuda N., Yin J. (2013). Process for Making CGRP Receptor Antagonists. Patent WO.

[38] Wood M.R., Bell I.M., Gallicchio S.N., Selnick H.G., Stump C.A., Zartman C.B. (2007). Substituted Spirocyclic CGRP Receptor Antagonists. Patent WO.

[39] Keam S.J. (2021). Piflufolastat F 18: Diagnostic First Approval. Mol. Diagn. Ther..

[40] Chen Y., Pullambhatla M., Foss C.A., Byun Y., Nimmagadda S., Senthamizhchelvan S., Sgouros G., Mease R.C., Pomper M.G. (2011). 2-(3-{1-Carboxy-5-[(6-[18F]fluoro-pyridine-3-carbonyl)-amino]-pentyl}-ureido)-pentanedioic acid, [18F]DCFPyL, a PSMA-based PET imaging agent for prostate cancer. Clin. Cancer Res. Off. J. Am. Assoc. Cancer Res..

[41] Olberg D.E., Arukwe J.M., Grace D., Hjelstuen O.K., Solbakken M., Kindberg G.M., Cuthbertson A. (2010). One Step Radiosynthesis of 6-[18F]Fluoronicotinic Acid 2,3,5,6-Tetrafluorophenyl Ester ([18F]F-Py-TFP): A New Prosthetic Group for Efficient Labeling of Biomolecules with Fluorine-18. J. Med. Chem..

[42] Canon J., Rex K., Saiki A.Y., Mohr C., Cooke K., Bagal D., Gaida K., Holt T., Knutson C.G., Koppada N. (2019). The clinical KRAS(G12C) inhibitor AMG 510 drives anti-tumour immunity. Nature.

[43] Lanman B.A., Allen J.R., Allen J.G., Amegadzie A.K., Ashton K.S., Booker S.K., Chen J.J., Chen N., Frohn M.J., Goodman G. (2020). Discovery of a Covalent Inhibitor of KRASG12C (AMG 510) for the Treatment of Solid Tumors. J. Med. Chem..

[44] Lanman B.A., Chen J., Reed Anthony B., Cee Victor J., Liu L., Kopecky D.J., Lopez P., Wurz R.P., Nguyen T.T., Booker S. (2018). Kras G12C Inhibitors and Method of Using the Same. Patent US.

[45] Zhang L., Griffin D.J., Beaver M.G., Blue L.E., Borths C.J., Brown D.B., Caille S., Chen Y., Cherney A.H., Cochran B.M. (2022). Development of a Commercial Manufacturing Process for Sotorasib, a First-in-Class KRASG12C Inhibitor. Org. Process Res. Dev..

[46] Lunning M., Vose J., Nastoupil L., Fowler N., Burger J.A., Wierda W.G., Schreeder M.T., Siddiqi T., Flowers C.R., Cohen J.B. (2019). Ublituximab and umbralisib in relapsed/refractory B-cell non-Hodgkin lymphoma and chronic lymphocytic leukemia. Blood.

[47] Weiss M., Miskin H., Sportelli P.S., Vakkalanka K.V.S. (2014). Combination of Anti-CD20 Antibody and PI3 Kinase Selective Inhibitor. Patent WO.

[48] Follmann M., Ackerstaff J., Redlich G., Wunder F., Lang D., Kern A., Fey P., Griebenow N., Kroh W., Becker-Pelster E.-M. (2017). Discovery of the Soluble Guanylate Cyclase Stimulator Vericiguat (BAY 1021189) for the Treatment of Chronic Heart Failure. J. Med. Chem..

[49] Yuan S., Luo Y.Q., Zuo J.H., Liu H., Li F., Yu B. (2021). New drug approvals for 2020: Synthesis and clinical applications. Eur. J. Med. Chem..

[50] Ohsawa I., Honda D., Suzuki Y., Fukuda T., Kohga K., Morita E., Moriwaki S., Ishikawa O., Sasaki Y., Tago M. (2021). Oral berotralstat for the prophylaxis of hereditary angioedema attacks in patients in Japan: A phase 3 randomized trial. Allergy.

[51] Yahya El-Kattan Y.S.B. (2020). Crystalline Salts of a Plasma Kallikrein Inhibitor. Patent US.

[52] Dhillon S. (2020). Decitabine/Cedazuridine: First Approval. Drugs.

[53] Ferraris D., Duvall B., Delahanty G., Mistry B., Alt J., Rojas C., Rowbottom C., Sanders K., Schuck E., Huang K.-C. (2014). Design, Synthesis, and Pharmacological Evaluation of Fluorinated Tetrahydrouridine Derivatives as Inhibitors of Cytidine Deaminase. J. Med. Chem..

[54] Brown K., Dixey M., Weymouth-Wilson A., Linclau B. (2014). The synthesis of gemcitabine. Carbohydr. Res..

[55] Markham A. (2020). Pralsetinib: First Approval. Drugs.

[56] Subbiah V., Shen T., Terzyan S.S., Liu X., Hu X., Patel K.P., Hu M., Cabanillas M., Behrang A., Meric-Bernstam F. (2021). Structural basis of acquired resistance to selpercatinib and pralsetinib mediated by non-gatekeeper RET mutations. Ann. Oncol. Off. J. Eur. Soc. Med. Oncol..

[57] Brubaker J.D., Kim J.L., Wilson K.J., Wilson D., Di Pietro L.V. (2017). Inhibitors of Ret. Patent US.

[58] Markham A., Keam S.J. (2020). Selumetinib: First Approval. Drugs.

[59] Wallace E.M., Lyssikatos J.P., Hurley B.T., Marlow A.L. (2003). N3 Alkylated Benzimiadazole Derivates Ad MEK Inhibitors. Patent WO.

[60] Mu L., Jie C.V.M.L., Treyer V., Schibli R. (2021). Tauvid™: The First FDA-Approved PET Tracer for Imaging Tau Pathology in Alzheimer’s Disease. Pharmaceuticals.

[61] Xia C.F., Arteaga J., Chen G., Gangadharmath U., Gomez L.F., Kasi D., Lam C., Liang Q., Liu C., Mocharla V.P. (2013). [(18)F]T807, a novel tau positron emission tomography imaging agent for Alzheimer’s disease. Alzheimer’s Dement. J. Alzheimer’s Assoc..

[62] Attardo G., Lister-James J., Xiong H., Lim N. (2015). Compounds and Their Use for Preparation of Tau Imaging Agents and Tau Imaging Formulations. Patent WO.

[63] Patridge E., Gareiss P., Kinch M.S., Hoyer D. (2016). An analysis of FDA-approved drugs: Natural products and their derivatives. Drug Discov. Today.

[64] Yuan S., Yu B., Liu H.M. (2020). New drug approvals for 2019: Synthesis and clinical applications. Eur. J. Med. Chem..

[65] Mei H., Remete A.M., Zou Y., Moriwaki H., Fustero S., Kiss L., Soloshonok V.A., Han J. (2020). Fluorine-containing drugs approved by the FDA in 2019. Chin. Chem. Lett..

[66] Markham A. (2019). Alpelisib: First Global Approval. Drugs.

[67] André F., Ciruelos E., Rubovszky G., Campone M., Loibl S., Rugo H.S., Iwata H., Conte P., Mayer I.A., Kaufman B. (2019). Alpelisib for PIK3CA-Mutated, Hormone Receptor-Positive Advanced Breast Cancer. N. Engl. J. Med..

[68] Furet P., Guagnano V., Fairhurst R.A., Imbach-Weese P., Bruce I., Knapp M., Fritsch C., Blasco F., Blanz J., Aichholz R. (2013). Discovery of NVP-BYL719 a potent and selective phosphatidylinositol-3 kinase alpha inhibitor selected for clinical evaluation. Bioorg. Med. Chem. Lett..

[69] Caravatti G., Fairhutst R.A., Guagnano V., Imbach P., Furet P. (2009). Thiazole Derivate Used as PI 3 KINASE Inhibitors. Patent WO.

[70] Caravatti G., Fairhutst R.A., Guagnano V., Imbach P., Furet P. (2010). Organic Compounds. Patent WO.

[71] Beuckmann C.T., Suzuki M., Ueno T., Nagaoka K., Arai T., Higashiyama H. (2017). In Vitro and In Silico Characterization of Lemborexant (E2006), a Novel Dual Orexin Receptor Antagonist. J. Pharmacol. Exp. Ther..

[72] Beuckmann C.T., Ueno T., Nakagawa M., Suzuki M., Akasofu S. (2019). Preclinical in vivo characterization of lemborexant (E2006), a novel dual orexin receptor antagonist for sleep/wake regulation. Sleep.

[73] Yoshida Y., Naoe Y., Terauchi T., Ozaki F., Doko T., Takemura A., Tanaka T., Sorimachi K., Beuckmann C.T., Suzuki M. (2015). Discovery of (1R,2S)-2-{[(2,4-Dimethylpyrimidin-5-yl)oxy]methyl}-2-(3-fluorophenyl)-N-(5-fluoropyridin-2-yl)cyclopropanecarboxamide (E2006): A Potent and Efficacious Oral Orexin Receptor Antagonist. J. Med. Chem..

[74] Lamb Y.N. (2019). Pexidartinib: First Approval. Drugs.

[75] Ibrahim P.N., Jin M., Matsuura S. (2016). Synthesis of 1H-Pyrrolo[2,3-B]pyridin Derivates That Modulate Kinases. Patent WO.

[76] Dodick D.W., Lipton R.B., Ailani J., Lu K., Finnegan M., Trugman J.M., Szegedi A. (2019). Ubrogepant for the Treatment of Migraine. N. Engl. J. Med..

[77] Bell I.M., Fraley M.E., Gallicchio S.N., Ginnetti A., Mitchell H., Paone D.V., Wang S.D.D.C., Zartman C.B., Stevenson H.E. (2012). Piperidone Carbozamide CGRP Receptor Antagonists. Patent WO.

[78] Flick A.C., Leverett C.A., Ding H.X., McInturff E. (2020). Synthetic Approaches to New Drugs Approved during 2018. J. Med. Chem..

[79] Al-Salama Z.T. (2018). Apalutamide: First Global Approval. Drugs.

[80] Smith M.R., Saad F., Chowdhury S., Oudard S., Hadaschik B.A., Graff J.N., Olmos D., Mainwaring P.N., Lee J.Y., Uemura H. (2018). Apalutamide Treatment and Metastasis-free Survival in Prostate Cancer. N. Engl. J. Med..

[81] Clegg N.J., Wongvipat J., Joseph J.D., Tran C., Ouk S., Dilhas A., Chen Y., Grillot K., Bischoff E.D., Cai L. (2012). ARN-509: A novel antiandrogen for prostate cancer treatment. Cancer Res..

[82] Jung M., Sawyers E.C., Ouk S.L., Tran C., Wongvipat J. (2007). Androgen Receptor Modulator for the Treatment of Prostate Cancer and Androgen Receptor-Associated Disease. Patent WO.

[83] Hughes D.L. (2020). Review of Synthetic Routes and Crystalline Forms of the Antiandrogen Oncology Drugs Enzalutamide, Apalutamide, and Darolutamide. Org. Process Res. Dev..

[84] Heo Y.A. (2018). Baloxavir: First Global Approval. Drugs.

[85] Noshi T., Kitano M., Taniguchi K., Yamamoto A., Omoto S., Baba K., Hashimoto T., Ishida K., Kushima Y., Hattori K. (2018). In vitro characterization of baloxavir acid, a first-in-class cap-dependent endonuclease inhibitor of the influenza virus polymerase PA subunit. Antivir. Res..

[86] Takashita E., Morita H., Ogawa R., Nakamura K., Fujisaki S., Shirakura M., Kuwahara T., Kishida N., Watanabe S., Odagiri T. (2018). Susceptibility of Influenza Viruses to the Novel Cap-Dependent Endonuclease Inhibitor Baloxavir Marboxil. Front. Microbiol..

[87] Shibahara S.F., Nobuaki, Toshikatsu M. (2017). Method for Producing Substituted Polycyclic Derivate and Crystal of Same. Patent JP.

[88] Koelblinger P., Dornbierer J., Dummer R. (2017). A review of binimetinib for the treatment of mutant cutaneous melanoma. Future Oncol..

[89] Ascierto P.A., Schadendorf D., Berking C., Agarwala S.S., van Herpen C.M., Queirolo P., Blank C.U., Hauschild A., Beck J.T., St-Pierre A. (2013). MEK162 for patients with advanced melanoma harbouring NRAS or Val600 BRAF mutations: A non-randomised, open-label phase 2 study. Lancet Oncol..

[90] Chen J. (2016). Synthetic Method of Binimetinib. Patent CN.

[91] Colombier M.A., Molina J.M. (2018). Doravirine: A review. Curr. Opin. HIV AIDS.

[92] Côté B., Burch J.D., Asante-Appiah E., Bayly C., Bédard, Blouin L.M., Campeau L.C., Cauchon E., Chan M., Chefson A. (2014). Discovery of MK-1439, an orally bioavailable non-nucleoside reverse transcriptase inhibitor potent against a wide range of resistant mutant HIV viruses. Bioorganic Med. Chem. Lett..

[93] Burch J., Nguyen N., Li C.S., St-onge M., Gauvreau D., Cote B. (2011). Non-Nucleoside Reverse Transcriptase Inhibitors. Patent WO.

[94] Markham A. (2018). Fostamatinib: First Global Approval. Drugs.

[95] Pine P.R., Chang B., Schoettler N., Banquerigo M.L., Wang S., Lau A., Zhao F., Grossbard E.B., Payan D.G., Brahn E. (2007). Inflammation and bone erosion are suppressed in models of rheumatoid arthritis following treatment with a novel Syk inhibitor. Clin. Immunol..

[96] Felfer U., Giselbrecht K.-H., Wolberg M. (2011). Syntesis of N4-(2,2-Dimethyl-4-[(Dihydrogen Phosphonoxy]-3-Oxo-5-Pyrydo [1,4] Oxazin6-yl)-5-Fluoro-N2-(3,4,5,-Trimethoxyphenyl)-2,4-Pyrimidinediamine Disodium Salt. Patent WO.

[97] Dhillon S. (2018). Ivosidenib: First Global Approval. Drugs.

[98] Popovici-Muller J., Lemieux R.M., Artin E., Saunders J.O., Salituro F.G., Travins J., Cianchetta G., Cai Z., Zhou D., Cui D. (2018). Discovery of AG-120 (Ivosidenib): A First-in-Class Mutant IDH1 Inhibitor for the Treatment of IDH1 Mutant Cancers. ACS Med. Chem. Lett..

[99] Lemieux R.M., Popovici-Muller J., Travins J., Cai Z., Cui D., Zhou D. (2013). Therapeutically Active Compounds and Their Methods of Use. Patent WO.

[100] Hoy S.M. (2018). Talazoparib: First Global Approval. Drugs.

[101] Wang B., Chu D., Feng Y., Shen Y., Aoyagi-Scharber M., Post L.E. (2016). Discovery and Characterization of (8S,9R)-5-Fluoro-8-(4-fluorophenyl)-9-(1-methyl-1H-1,2,4-triazol-5-yl)-2,7,8,9-tetrahydro-3H-pyrido[4,3,2-de]phthalazin-3-one(BMN673,Talazoparib), a Novel, Highly Potent, and Orally Efficacious Poly(ADP-ribose) Polymerase-1/2 Inhibitor, as an Anticancer Agent. J. Med. Chem..

[102] Xu Y.Y., Peter W., Michael X., Douglas C. (2017). Synthesis of Parp Inhibitor Talazoparib. Patent US.

[103] Mospan C., Mospan G., Byland E., Whitaker W.B., Xiong L., Dunlap J., Canupp K. (2018). Drug updates and approvals: 2018 in review. Nurse Pract..

[104] Donaldson S.H., Pilewski J.M., Griese M., Cooke J., Viswanathan L., Tullis E., Davies J.C., Lekstrom-Himes J.A., Wang L.T. (2018). Tezacaftor/Ivacaftor in Subjects with Cystic Fibrosis and F508del/F508del-CFTR or F508del/G551D-CFTR. Am. J. Respir. Crit. Care Med..

[105] Hughes D.L. (2019). Patent Review of Synthetic Routes and Crystalline Forms of the CFTR-Modulator Drugs Ivacaftor, Lumacaftor, Tezacaftor, and Elexacaftor. Org. Process Res. Dev..

[106] Tanoury G.J., Harrison C., Littler B.J., Rose P.J., Hughes R.M., Jung Y.C., Siesel D.A., Lee E.C., Belmont D.T. (2011). Process of Producing Cycloalkylcarboxamido-Indole Compounds. Patent WO.

[107] De la Torre B.G., Albericio F. (2018). The Pharmaceutical Industry in 2017. An Analysis of FDA Drug Approvals from the Perspective of Molecules. Molecules.

[108] Mullard A. (2018). 2017 FDA drug approvals, Nature reviews. Drug Discov..

[109] Martin M., Garcia-Saenz J.A. (2020). Abemaciclib, a CDK4 and CDK6 inhibitor for the treatment of metastatic breast cancer. Futur. Oncol..

[110] Frederick M.O., Kjell D.P. (2015). A synthesis of abemaciclib utilizing a Leuckart–Wallach reaction. Tetrahedron Lett..

[111] Frederick M.O., Pietz M.A., Kjell D.P., Richey R.N., Tharp G.A., Touge T., Yokoyama N., Kida M., Matsuo T. (2017). Development of a Leuckart–Wallach Reaction in Flow for the Synthesis of Abemaciclib. Org. Process Res. Dev..

[112] Markham A. (2017). Delafloxacin: First Global Approval. Drugs.

[113] Harnett S.J., Fraise A.P., Andrews J.M., Jevons G., Brenwald N.P., Wise R. (2004). Comparative study of the in vitro activity of a new fluoroquinolone, ABT-492. J. Antimicrob. Chemother..

[114] Barnes D.M., Christesen A.C., Engstrom K.M., Haight A.R., Hsu M.C., Lee E.C., Peterson M.J., Plata D.J., Raje P.S., Stoner E.J. (2006). Chlorination at the 8-Position of a Functionalized Quinolone and the Synthesis of Quinolone Antibiotic ABT-492. Org. Process Res. Dev..

[115] Kim E.S. (2017). Enasidenib: First Global Approval. Drugs.

[116] Stein E.M., DiNardo C.D., Pollyea D.A., Fathi A.T., Roboz G.J., Altman J.K., Stone R.M., DeAngelo D.J., Levine R.L., Flinn I.W. (2017). Enasidenib in mutant IDH2 relapsed or refractory acute myeloid leukemia. Blood.

[117] García-Llinás X., Bauzá A., Seth S.K., Frontera A. (2017). Importance of R–CF3···O Tetrel Bonding Interactions in Biological Systems. J. Phys. Chem. A.

[118] Cianchetta B.D.G., Popovici-Muller J.F., Salituro G., Saunders J.O., Travins J., Yan S., Guo T., Zhang L. (2013). Therapeutically Active Compounds and Their Methods of Use. Patent WO.

[119] Lamb Y.N. (2017). Glecaprevir/Pibrentasvir: First Global Approval. Drugs.

[120] Lawitz Eric J., O’Riordan William D., Asatryan A., Freilich Bradley L., Box Terry D., Overcash J.S., Lovell S., Ng Teresa I., Liu W., Campbell A. (2016). Potent Antiviral Activities of the Direct-Acting Antivirals ABT-493 and ABT-530 with Three-Day Monotherapy for Hepatitis C Virus Genotype 1 Infection. Antimicrob. Agents Chemother..

[121] Cink R.D., Lukin K.A., Bishop R.D., Zhao G., Pelc M.J., Towne T.B., Gates B.D., Ravn M.M., Hill D.R., Ding C. (2020). Development of the Enabling Route for Glecaprevir via Ring-Closing Metathesis. Org. Process Res. Dev..

[122] Kallemeyn J.M., Engstrom K.M., Pelc M.J., Lukin K.A., Morrill W.H., Wei H., Towne T.B., Henle J., Nere N.K., Welch D.S. (2020). Development of a Large-Scale Route to Glecaprevir: Synthesis of the Macrocycle via Intramolecular Etherification. Org. Process Res. Dev..

[123] Kim E.S. (2018). Letermovir: First Global Approval. Drugs.

[124] Goldner T., Hewlett G., Ettischer N., Ruebsamen-Schaeff H., Zimmermann H., Lischka P. (2011). The novel anticytomegalovirus compound AIC246 (Letermovir) inhibits human cytomegalovirus replication through a specific antiviral mechanism that involves the viral terminase. J. Virol..

[125] Humphrey G.R., Dalby S.M., Andreani T., Xiang B., Luzung M.R., Song Z.J., Shevlin M., Christensen M., Belyk K.M., Tschaen D.M. (2016). Asymmetric Synthesis of Letermovir Using a Novel Phase-Transfer-Catalyzed Aza-Michael Reaction. Org. Process Res. Dev..

[126] Chung C.K., Liu Z., Lexa K.W., Andreani T., Xu Y., Ji Y., DiRocco D.A., Humphrey G.R., Ruck R.T. (2017). Asymmetric Hydrogen Bonding Catalysis for the Synthesis of Dihydroquinazoline-Containing Antiviral, Letermovir. J. Am. Chem. Soc..

[127] Wang P.S., Shen M.L., Wang T.C., Lin H.C., Gong L.Z. (2017). Access to Chiral Hydropyrimidines through Palladium-Catalyzed Asymmetric Allylic C-H Amination. Angew. Chem..

[128] Ng Teresa I., Krishnan P., Pilot-Matias T., Kati W., Schnell G., Beyer J., Reisch T., Lu L., Dekhtyar T., Irvin M. (2017). In Vitro Antiviral Activity and Resistance Profile of the Next-Generation Hepatitis C Virus NS5A Inhibitor Pibrentasvir. Antimicrob. Agents Chemother..

[129] Degoey D.A., Kati W.M., Hutchins C.W., Donner P.L., Krueger A.C., Randolph J.T., Motter C.E., Nelson L.T., Patel S.V., Matulenko M.A. (2012). Anti-Viral Compounds. Patent WO.

[130] Heo Y.-A., Deeks E.D. (2018). Sofosbuvir/Velpatasvir/Voxilaprevir: A Review in Chronic Hepatitis C. Drugs.

[131] Bjornson K., Canales E., Cotell J., Karki J.K., Katana K.A., Kato A.D., Kobayashi T., Link J.O., Martinez R., Phillips B. (2014). Inhibitors of Hepatitis C Virus. Patent WO.

[132] Mullard A. (2017). 2016 FDA drug approvals. Nat. Rev. Drug Discov..

[133] Musella A., Bardhi E., Marchetti C., Vertechy L., Santangelo G., Sassu C., Tomao F., Rech F., D’Amelio R., Monti M. (2018). Rucaparib: An emerging parp inhibitor for treatment of recurrent ovarian cancer. Cancer Treat. Rev..

[134] Thomas H.D., Calabrese C.R., Batey M.A., Canan S., Hostomsky Z., Kyle S., Maegley K.A., Newell D.R., Skalitzky D., Wang L.-Z. (2007). Preclinical selection of a novel poly(ADP-ribose) polymerase inhibitor for clinical trial. Mol. Cancer Ther..

[135] Webber S.E., Canan-Koch S.S., Tikhe J.G., Thoresen L.H. (2000). Tricyclic Inhibitors of Poly(ADP-Ribose) Polymerases. Patent WO.

[136] Bonaventura A., Montecucco F. (2016). Sofosbuvir/velpatasvir: A promising combination. World J. Hepatol..

[137] Keating G.M., Vaidya A. (2014). Sofosbuvir: First Global Approval. Drugs.

[138] Asselah T. (2014). Sofosbuvir for the treatment of hepatitis C virus. Expert Opin. Pharmacother..

[139] Sofia M.J., Bao D., Chang W., Du J., Nagarathnam D., Rachakonda S., Reddy P.G., Ross B.S., Wang P., Zhang H.-R. (2010). Discovery of a β-d-2′-Deoxy-2′-α-fluoro-2′-β-C-methyluridine Nucleotide Prodrug (PSI-7977) for the Treatment of Hepatitis C Virus. J. Med. Chem..

[140] Wang P., Chun B.-K., Rachakonda S., Du J., Khan N., Shi J., Stec W., Cleary D., Ross B.S., Sofia M.J. (2009). An Efficient and Diastereoselective Synthesis of PSI-6130: A Clinically Efficacious Inhibitor of HCV NS5B Polymerase. J. Org. Chem..

